# Exposures in Indoor Air Affecting Health

**DOI:** 10.1111/all.70179

**Published:** 2025-12-04

**Authors:** Maria Hartiala, Varpu Elenius, Alicia Aguado Pesquera, Silas Androulakis, Isabella Annesi‐Maesano, Artur Badyda, Sicco Brandsma, Ioanna Chatziprodromidou, Goran Gajski, Judith Garcia‐Aymerich, Chiara Giorio, Timo Hugg, Jouni J. K. Jaakkola, Sarah Koch, Pim E. G. Leonards, Angeliki Matrali, Lisa Melymuk, Natalie Mueller, Adam Muszyński, Jet Opbroek, Inês Paciência, Spyros N. Pandis, Sofya Pozdniakova, Aino K. Rantala, Sandra Rodríguez Sufuentes, Linda Schenk, Eva Sugeng, Adrià Sunyer‐Caldú, Apostolos Vantarakis, Alexander Zherebker, Ernesto Alfaro‐Moreno, Pernilla Bohlin Nizzetto, José Fermoso Dominguez, Stylianos Karatzas, Francesco Mureddu, Heidi Salonen, Nikolaos Papadopoulos, Tuomas Jartti, the IDEAL Consortium

**Affiliations:** ^1^ Department of Pediatrics and Adolescent Medicine Turku University Hospital and University of Turku Turku Finland; ^2^ CARTIF Technology Center Boecillo Spain; ^3^ Foundation for Research and Technology Hellas (FORTH) and University of Patras Patras Greece; ^4^ Department of Chemical Engineering University of Patras Patras Greece; ^5^ University Hospital Institute Immune4Cure INSERM and University of Montpellier Montpellier France; ^6^ Division of Respiratory Medicine, Allergology, and Thoracic Oncology University Hospital of Montpellier Montpellier France; ^7^ Warsaw University of Technology Warsaw Poland; ^8^ Amsterdam Institute for Life and Environment (A‐LIFE) Vrije Universiteit Amsterdam Amsterdam the Netherlands; ^9^ Department of Public Health, Medical School University of Patras Patras Greece; ^10^ Division of Toxicology Institute for Medical Research and Occupational Health Zagreb Croatia; ^11^ Barcelona Institute for Global Health (ISGlobal) Barcelona Spain; ^12^ Universitat Pompeu Fabra (UPF) Barcelona Spain; ^13^ CIBER Epidemiología y Salud Pública (CIBERESP) Madrid Spain; ^14^ Yusuf Hamied Department of Chemistry University of Cambridge Cambridge UK; ^15^ Center for Environmental and Respiratory Health Research (CERH), Research Unit of Population Health University of Oulu Oulu Finland; ^16^ Finnish Meteorological Institute Helsinki Finland; ^17^ RECETOX, Faculty of Science Masaryk University Brno Czechia; ^18^ Institute of Environmental Medicine Karolinska Institutet Stockholm Sweden; ^19^ Department of Environmental Science, Exposure and Effects Unit, Science for Life Laboratory Stockholm University Stockholm Sweden; ^20^ International Iberian Nanotechnology Laboratory Braga Portugal; ^21^ Department of Environmental Chemistry and Health Effects NILU Kjeller Norway; ^22^ Department of Civil Engineering University of Patras Patras Greece; ^23^ The Lisbon Council Brussels Belgium; ^24^ Department of Civil Engineering Aalto University Espoo Finland; ^25^ International Laboratory for Air Quality and Health (WHO CC for Air Quality and Health) Queensland University of Technology (QUT) Brisbane Australia; ^26^ Allergy Department, 2nd Pediatric Clinic University of Athens Athens Greece; ^27^ Lydia Becker Institute of Immunology University of Manchester Manchester UK

**Keywords:** exposure, health, indoor air quality, pollution

## Abstract

Indoor air quality (IAQ) is influenced by a wide range of chemical, biological and physical agents that can negatively impact physical, immunological and mental health. Adverse health effects depend on the type and concentration of pollutants, duration of exposure, and individual susceptibility. The availability of data on IAQ is limited, as are standardized approaches for evaluating its health impact. This expert review aims to describe the most important indoor air determinants affecting health, and present the IDEAL cluster, which comprises seven EU‐funded scientific projects on the topic of IAQ and human health. Across the IDEAL projects, knowledge is generated on exposure to a wide range of indoor air pollutants, including well‐known hazards and more explorative chemical and microbiological determinants. The projects will also contribute to the implementation of low‐cost and/or real‐time sensors on IAQ, as well as advanced chemical and microbiological analyses, and evaluate various interventions to improve IAQ. Several of them focus on particularly vulnerable groups. Raising public awareness and implementing measures to reduce pollutant levels are essential for safeguarding health, particularly in urban areas with elevated pollution levels.

**Abbreviations**
BPA: bisphenol A
CH_4_
: methane
CO: carbon monoxide
CO_2_
: carbon dioxide
COPD: chronic obstructive pulmonary disease
ETS: environmental tobacco smoke
H_2_S: hydrogen sulfide
IAQ: indoor air quality
NH_3_
: ammonia
NO_2_
: nitrogen dioxide
NOx: nitrogen oxides
O_3_
: ozone
PAH: polycyclic aromatic hydrocarbon
PCB: polychlorinated biphenyl
PFAS: polyfluoroalkyl substance
PM: particulate matter
RH: relative humidity
RSV: respiratory syncytial virus
RV: rhinovirus
SO_2_
: sulfur dioxide
SVOC: semi‐volatile organic compound
T: temperature
TVOC: total volatile organic compounds
UFP: ultrafine particle
VO: Crvolatile organic compound

## 1 Introduction

Indoor air quality (IAQ) and health are closely connected. People spend approximately 90% of their time indoors [1]. Uncontaminated indoor air sustains cognition and working capacity, reduces the spread of infectious and allergic agents, protects against pollutants, and strengthens the immune system when biodiversified [2, 3]. Conversely, low‐quality indoor air, which contains a wide range of chemical, biological, and physical agents, can cause adverse physical, immunological and mental health effects, temporarily or permanently [4, 5, 6]. Beyond the known hazards, we are faced with a large amount of uncertainty in the composition and potential impacts of chemicals in indoor environments. Today, > 350,000 chemicals are in commerce [7], and many of these chemicals are used in consumer products and building materials with the potential to be released into indoor environments, with further possibility of reaction and degradation [8]. Air pollution contributes to approximately 400,000 premature deaths and millions of disability‐adjusted life years annually in Europe [9, 10]. The economic burden related to air pollution in Europe is estimated at 100–200 billion euros per year, consisting of healthcare expenses, lost work productivity, and reduced quality of life [11].

**FIGURE 1:**
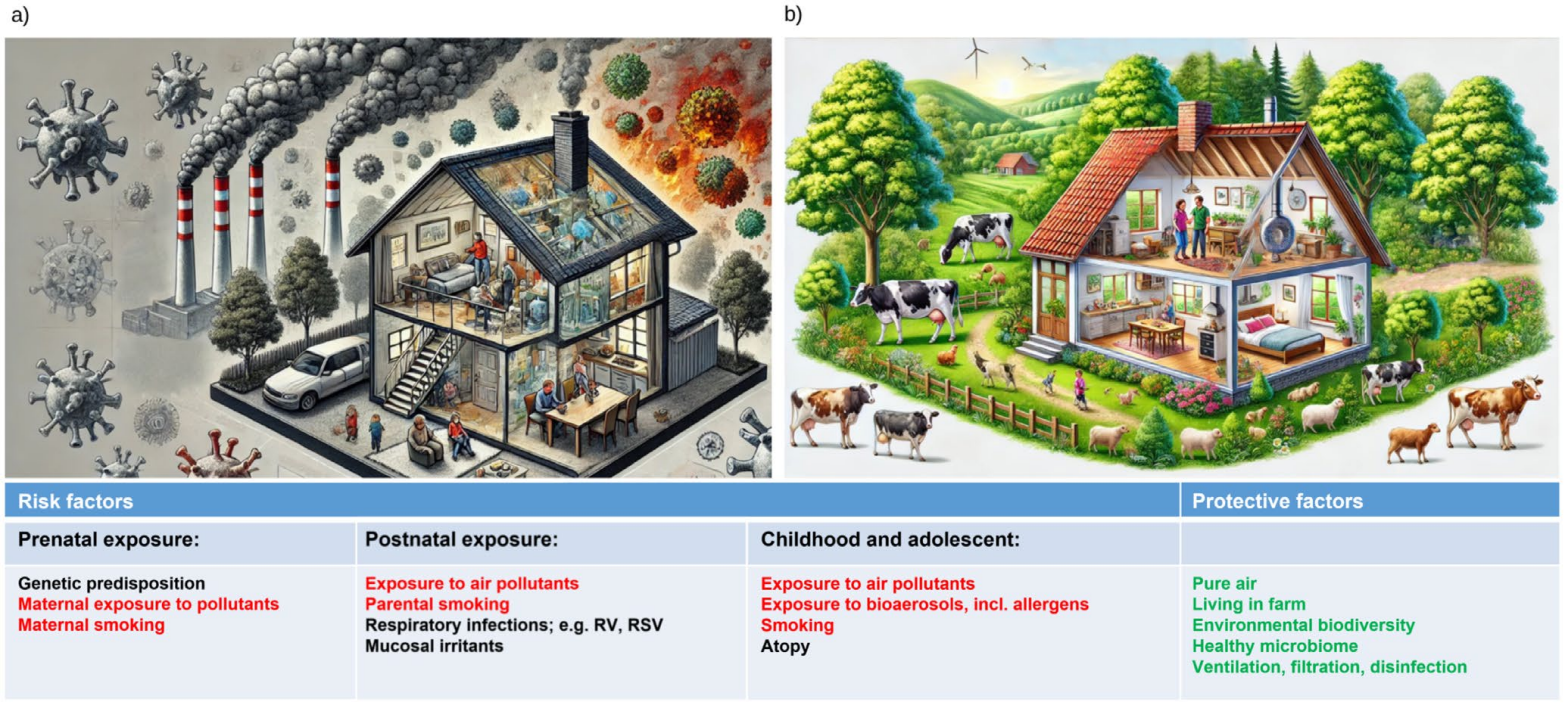
Risk factors and protective factors related to the health effects of air quality. Avoidable factors are shown in red, protective factors in green and others in black. The figures depict (a) poor and (b) good air quality (modified after creation by ChatGPT). RSV, respiratory syncytial virus; RV, rhinovirus.

The manifestation of health effects depends on both the magnitude of exposure and people's susceptibility (Figure 1). The most vulnerable populations to the adverse effects of indoor air pollution include children, chronically ill individuals, and the elderly [3, 12, 13, 14]. Exposure to pollutants begins during the prenatal period when the fetus is exposed to pollutants while in utero. Children are particularly at risk due to several factors: their developing respiratory and immune systems, the ratio of smaller body size and inhaled air volume, and their longer life expectancy in which the risk is expressed. Additionally, children's increased physical activity and exploratory behavior (e.g., hand‐to‐mouth activity), higher breathing rate, tendency to breathe through their mouths, and frequent interaction with ground‐level pollutants further heighten their exposure to harmful substances [3, 15].

Complex interactions between environmental factors and dynamic biological processes remain insufficiently understood, posing a challenge in developing effective strategies for monitoring and improving IAQ. The IDEAL cluster comprising seven EU‐funded scientific projects (EDIAQI, InChildHealth, INQUIRE, K‐HEALTHinAIR, LEARN, SynAir‐G, TwinAIR) addresses this need by focusing on both indoor and outdoor air pollution, their main sources and their effects on public health across Europe (Tables 1 and 2). The cluster's goal is to optimize synergies, avoid research overlaps and maximize the impact of these projects. In this expert review, we describe both chemical and biological pollutants related to air quality and the relation to human health.

**TABLE 1:** The IDEAL cluster projects.

Project	Aim	Country	Setting	Monitoring and sampling
EDIAQI	To validate user‐friendly IAQ monitoring solutions that can help create a long‐term Europe‐wide knowledge base for risk factors associated with standard and novel indoor air pollutants	Belgium, Austria, Croatia, Denmark, Estonia, Germany, Greece, Italy, Lithuania, Slovenia, Spain	Kindergarden, school, social care building, office, entertainment, residential building; focus on pollution sources, toxicology and health risks (200 children)	Monitoring: low‐cost sensor, active sampling with pumps on the filter, passive sampling with solid‐state nuclear track detector, passive sampling with activated charcoal filters, passive sampling on filters, passive sampling with Radiello air quality monitoring system, active sampling on adsorption tubes with pumps Biomarkers of effect: alkaline comet assay, cytokinesis‐block micronucleus assay, buccal micronucleus assay, FeNO Biomarkers of exposure: inductively coupled plasma MS Microbiological analysis: analysis of dust samples to retrieve microbiome data from children's beddings Prospective cohort: demographic data, extensive clinical data, transcriptomics, biological samples and follow‐up data
InChildHealth	To identify determinants for IAQ and evaluate their health impact in environments occupied by school children, focusing on chemicals, particle concentrations, microorganisms and physical parameters	Finland, Austria, Denmark, Greece, Portugal, Spain, Switzerland, United Kingdom	School, home, sports hall, transport; 50 primary schools (≈4500 school children invited for the epidemiological study)	Monitoring: real‐time monitoring solutions for gaseous and particulate pollutants and organisms such as bacteria and fungi, multi‐methods based on GC with tandem MS/MS and LC for non‐polar and polar compounds Microbiological analysis: passive samplers (settled dust, swab, electrostatic dust collector) and active sampling (impinger and impaction), microbial assessment by culture‐dependent and independent methods, microbial resistance profile, mycotoxins and endotoxins, standard methods for microbiology and inflammation marker detection Subjective assessment: questionnaires
INQUIRE	To evaluate innovative actions to reduce hazardous chemical and biological determinants in homes, positively impacting the health of residents, focusing particularly on infants and young children (< 5 years old)	Norway, Australia, Belgium, Czech Republic, Estonia, Finland, Germany, Italy, the Netherlands, Portugal, Slovenia, Sweden, United Kingdom	Home of small children; 200 homes	Monitoring: low‐cost sensors, passive air sampling with Tenax Tubes for VOCs, passive air sampling with PDMS for SVOCs, settled dust collection for SVOCs and in vitro analyses, collection of household products for SVOCs, in vitro and in vivo bioassays Chemical characterization: broad‐scale screening of VOCs in indoor and outdoor air (HRGC–MS), broad‐scale screening of SVOCs in indoor and outdoor air (HRLC–MS, HRGC–MS), broad‐scale screening of SVOCs in settled dust and products (HRLC‐MS, HRGC–MS), emission of VOCs from products (GC‐MS) Biomarkers of exposure: targeted analyses of metabolites in urine (LC–MS/MS) Biological characterization: standard methods for endotoxins, microbials and allergens in settled dust Subjective assessment: questionnaires
K‐HEALTHinAIR	To increase knowledge about chemical and biological indoor air pollutants affecting human health, and to provide solutions for more accurate monitoring and improvement of IAQ	Spain, Austria, Germany, Ireland, the Netherlands Norway, Poland, Portugal	Hospital, metro station, market, senior home, canteen, students' residence, lecture hall, home, school; focus on pollution sources	Monitoring: active and passive air samplers, for VOCs, and aldehydes, low‐cost sensors, aspirators (quartz filters and sorbent tubes), microbiological samplers (impactors, impingers), gravimetry (PM), HRMS (VOCs), GC‐FID (PAHs) Microbiological analysis: culture‐dependent techniques, 16S rRNA and internal transcribed spacer rRNA amplicons sequencing
LEARN	To control and evaluate IAQ at schools and its impact on children's health and cognition	Denmark, Belgium, Germany, Greece, the Netherlands, Switzerland, Portugal, Spain	School; focus on sensors	Monitoring: active and passive air samplers, for VOCs, and aldehydes, low‐cost sensors for detecting the levels of PM, and passive and active samplers for PM to characterize the PMs, GC‐FID, GC–MS and high‐performance LC, scanning electron microscopy, transmission electron microscopy Microbiological analysis: standard bacteria and fungi methods
SynAir‐G	To reveal and quantify synergistic interactions between different pollutants affecting health, from mechanisms to real life, focusing on the school setting	Greece, Finland, France, Georgia, United Kingdom	School; 2000 children, prospectively followed for a school year	Monitoring: HR aerosol MS, proton transfer reaction, MS, scanning mobility particle sizer, aethalometer, gas‐monitors, ENSENSIA‐air quality monitoring station, automated bioaerosol counts (Pollensense) Microbiological analysis: devices sensing biological pollutants, biologic samples (blood, nasal secretion, urine samples) Subjective assessment: questionnaires Objective health assessment: spirometry (lung function), microbiome
TwinAIR	To explore the impacts of indoor environments on acute and chronic health and wellbeing outcomes (including respiratory health, general symptoms, mental health, somatization, productivity), and the interaction between air quality and the human microbiome, and to provide practical tools to mitigate IAQ risks in urban settings	Spain, Germany, Greece, Sweden, United Kingdom	Workplace, university lecture hall, study area, library, hospital, elderly care centre, public transport (bus); 900 adults, across 45 diverse indoor spaces used for work, study, leisure or travel	Monitoring: low‐cost continuously measuring sensors, high volume sampler (Sibata, quartz filters) and MD8 Airport (Sartorius, gelatin filters), elemental composition analysis using inductively coupled plasma atomic emission spectroscopy and ‐MS Microbiological analysis: metagenomics shotgun sequencing, culturomics and antibiotic resistance testing Subjective assessment: questionnaires Objective health assessment: spirometry (lung function), microbiome and resistome

Abbreviations: FeNO, fractional exhaled nitric oxide; FID, flame ionization detector; GC, gas chromatography; HR, high resolution; IAQ, indoor air quality; LC, liquid chromatography; MS, mass spectrometry; PAH, polycyclic aromatic hydrocarbon; PM, particulate matter; SVOC, semi‐volatile organic compound; VOC, volatile organic compound.

**TABLE 2:** Indoor exposures investigated by the IDEAL cluster projects.

Indoor exposures	EDIAQI	InChildHealth	INQUIRE	K‐HEALTHinAIR	LEARN	SynAir‐G	TwinAIR
PM_1_, PM_2.5_, PM_10_	×	×	×	×	×	×	×
CO, CO_2_	×	×	×	×	×	×	×
NO, NO_2_, NOx	×	×				×	×
VOCs, SVOCs, TVOCs	×	×	×	×	×	×	×
PAHs	×		×	×	×		
O_3_	×	×				×	×
SO_2_		×				×	
NH_3_, CH_4_, H_2_S		×					
Flame retardants, PFASs, phthalates, biocides		×	×				
Aldehydes	×			×	×	×	×
Radon	×	×		×			×
Metals	×		×				×
Microplastics	×						
Allergens		×	×			×	
Microbes (viruses, bacteria, fungi)	×	×	×	×	×	×	×
Temperature, humidity	×	×	×	×	×	×	×
Noise, light				×			×

Abbreviations: CH_4_, methane; CO, carbon monoxide; CO_2_, carbon dioxide; H_2_S, hydrogen sulphide; NH_3_, ammonia; NO, nitric oxide; NO_2_, nitrogen dioxide; NOx, nitrogen oxides; O_3_, ozone; PAH, polycyclic aromatic hydrocarbon; PFAS, polyfluoroalkyl substance; PM_x_, particulate matter with aerodynamic diameter less than or equal to × μm; SO_2_, sulfur dioxide; SVOC, semi‐volatile organic compound; TVOC, total volatile organic compounds; VOC, volatile organic compound.

## 2 Indoor Exposure

### 2.1 Gaseous Compounds

**FIGURE 2:**
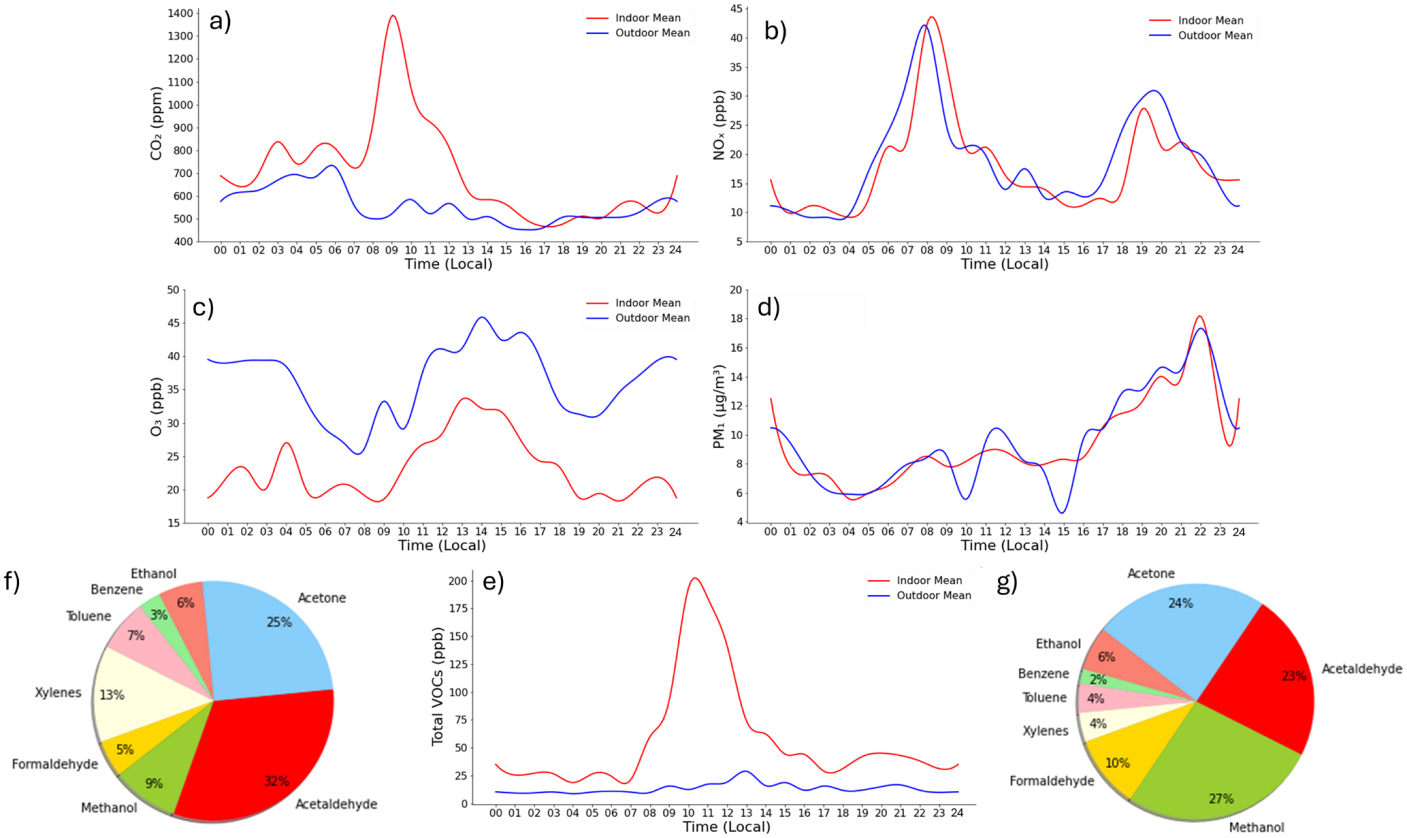
Typical variation of concentrations of major indoor air pollutants in a classroom during the day. (a) CO_2_, (b) NO_x_, (c) O_3_, (d) PM_1_, (e) total measured VOCs, (f) percent contribution of each VOC component to the total VOCs based on carbon, (g) molecular percent contribution of each VOC component to the total VOCs. The measurements were performed during the SynAirG project in a typical Athens elementary school during January 2024. CO_2_, carbon dioxide; NO_x_, nitrogen oxides; O_3_, ozone; PM_1_, particulate matter with aerodynamic diameter less than or equal to 1 μm; VOC, volatile organic compound.

**FIGURE 3:**
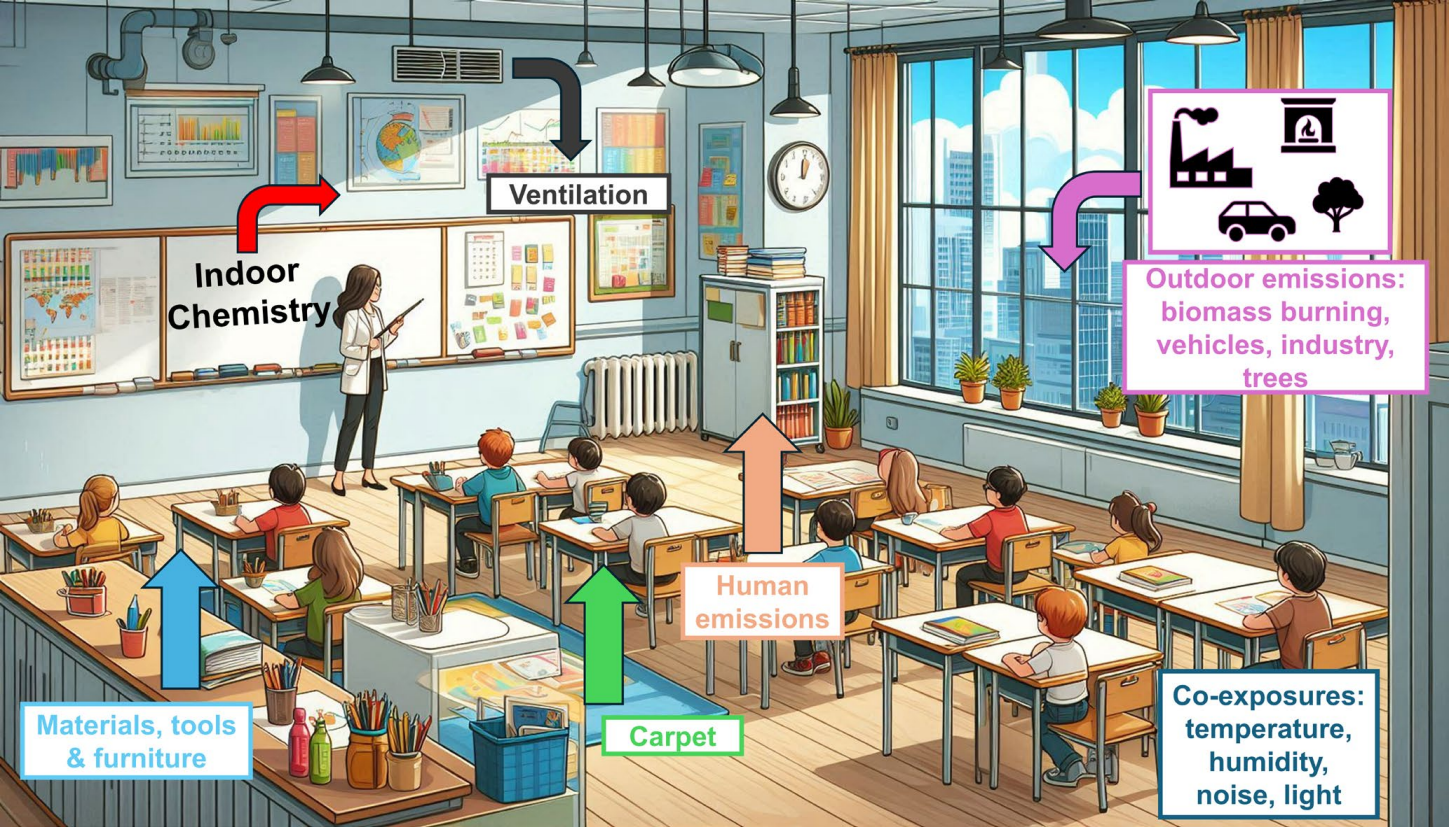
Schematic drawing of the different sources (colored arrows) of indoor air pollutants in classrooms: Outdoor to indoor, chemical emissions from surfaces, and materials. Indoor environmental quality is also affected by co‐exposures, such as temperature, humidity, noise, light and the synergies between them and occupation density. The classroom is drawn by ChatGPT.

Carbon monoxide (CO) and dioxide (CO_2_), nitrogen dioxide (NO_2_), sulfur dioxide (SO_2_), ozone (O_3_), and other gases significantly worsen IAQ [8, 14, 16, 17], as shown in Figure 2. In addition to indoor sources, outdoor air‐related emissions from transportation, domestic heating, biomass burning, industry and others influence indoor levels of these pollutants (Figure 3).

Indoor CO ranges from 0.5 to 5 ppm but surpasses 30 ppm when using gas stoves [18] or other sources involving incomplete combustion like unvented kerosene heaters or smoking [14, 19, 20, 21]. An insufficient supply of replacement air in relation to the number of users can also raise the CO level considerably. The risk of incomplete combustion is high in solid fuel stoves, especially if their technical condition is poor and the room is poorly ventilated.

CO_2_ is a natural component of indoor air, primarily produced by human respiration [14, 22]. Inadequate ventilation increases CO_2_ concentrations. Levels above 1000 ppm have been connected to health effects first appearing as neurological symptoms and impaired cognitive function [22, 23].

NO_2_ is primarily emitted indoors by combustion appliances like gas stoves and heaters and outdoors by the burning of fuel [14, 16, 18, 19, 20, 21]. SO_2_ is mainly emitted during the combustion of sulfur‐containing fossil fuels, such as coal and oil [16, 19, 20]. More than half is emitted by industrial activities, but it is also released by residential and business heating. Photochemically formed O_3_ infiltrates indoor environments [18, 20, 24]. Typical indoor concentrations range from 20% to equal outdoor levels, with increased levels during appliance use [18]. Exposure to these gases causes respiratory irritation and asthma‐related symptoms [17, 18, 19, 20].

### 2.2 Particulate Matter, Polycyclic Aromatic Hydrocarbons, Metals and Microplastics

Particulate matter (PM) refers to all airborne particles. More specifically PM_x_ refers to the particle mass for all particles with diameters not exceeding × μm. PM_2.5_ is considered the most relevant fraction for human health and is the major focus of legislation. Many households in low‐ and middle‐income countries exceed WHO guidelines of indoor PM_2.5_ levels (24‐h ≤ 15 μg/m^3^, annual ≤ 5 μg/m^3^), for example, in South Asia and Sub‐Saharan Africa average indoor PM_2.5_ levels may frequently exceed 200–300 μg/m^3^, while European levels are generally below 30 μg/m^3^ [25].

**FIGURE 4:**
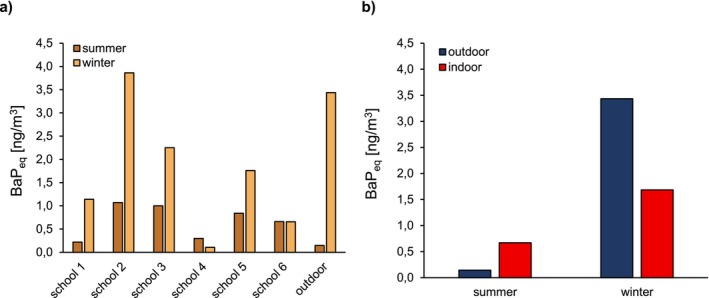
Marked variations in benzo(a)pyrene equivalent (BaP_eq_) concentrations in 6 schools in the Warsaw area (Poland) in summer and winter seasons (a) and mean values for all schools in both seasons (b). Continuous samples of PM_4_ were collected on quartz filters (GilAir Plus aspirators, Sensidyne, USA) within a 7‐day period in different schools and PAHs were determined using a gas chromatograph. Outdoor samples were collected in the city, near residential blocks heated by the municipal heating system and single‐family houses using solid fuels. Buildings using solid fuels for heating significantly increase the concentrations of PAHs in the air during the winter season. Consequently, interiors of neighboring buildings (even those heated in a different way), like nearby schools, experience elevated PAH concentrations in winter (schools 2, 3 and 5). Buildings located further away from residential sources are usually less affected by high PAH concentrations (schools 1, 4 and 6). Samples were collected in summer 2023 and winter 2023/2024 as part of the K‐HEALTHinAIR project. PAH, polycyclic aromatic hydrocarbon; PM_4_, particulate matter with aerodynamic diameter less than or equal to 4 μm.

A major source of indoor PM is outdoor air [26, 27, 28]. Road traffic, domestic heating, biomass burning, industry, dust and pollen (birch and grasses) are some of the significant outdoor sources of PM [14]. Indoor PM originates from biological sources, such as pet dander and dust mites, as well as human activities, including cooking, heating with stoves and fireplaces, burning candles, cigarettes, and incense, along with the use of chalk in classrooms [14, 16, 20, 21, 29, 30]. Additionally, cleaning products, laundry detergents and air fresheners emit indoor aerosol pollutants. Environmental tobacco smoke (ETS) remains a critical source of indoor pollutants in homes despite public smoking bans [31]. ETS, burning of fuels for heating, and candle emissions are also major sources of polycyclic aromatic hydrocarbons (PAHs) [14, 32]. Heating buildings using solid fuels significantly increases the concentrations of PAHs in the air during the winter season, and buildings located further away from residential sources are usually less affected by high PAH concentrations, as shown in Figure 4.

Particle‐bound metals can be produced during combustion processes [33] (e.g., cooking [34], smoking [35], candle and incense burning [36]), or by using consumer products, electrical components and building materials (e.g., paint). Notable examples are iron [35, 36, 37], copper [36, 37, 38], zinc [35, 36, 38], manganese [36], cadmium, often associated with cigarette smoke and combustion activities [35], and lead [33, 35], linked to historical use in paints, pipes, and combustion processes. Personal care products can contain metals and contaminants [36, 39, 40]. Microplastics, plastic particles less than 5 mm in size, are also of emerging concern due to their potential to cause respiratory and systemic health effects [41].

### 2.3 Volatile Organic Compounds

Volatile organic compounds (VOCs) are defined by their property of having a high vapor pressure so that under normal indoor conditions they can evaporate and are present in indoor air as gases [14, 42, 43]. VOCs are not defined by their toxicological properties. Nevertheless, many VOCs can pose a risk to human health.

**FIGURE 5:**
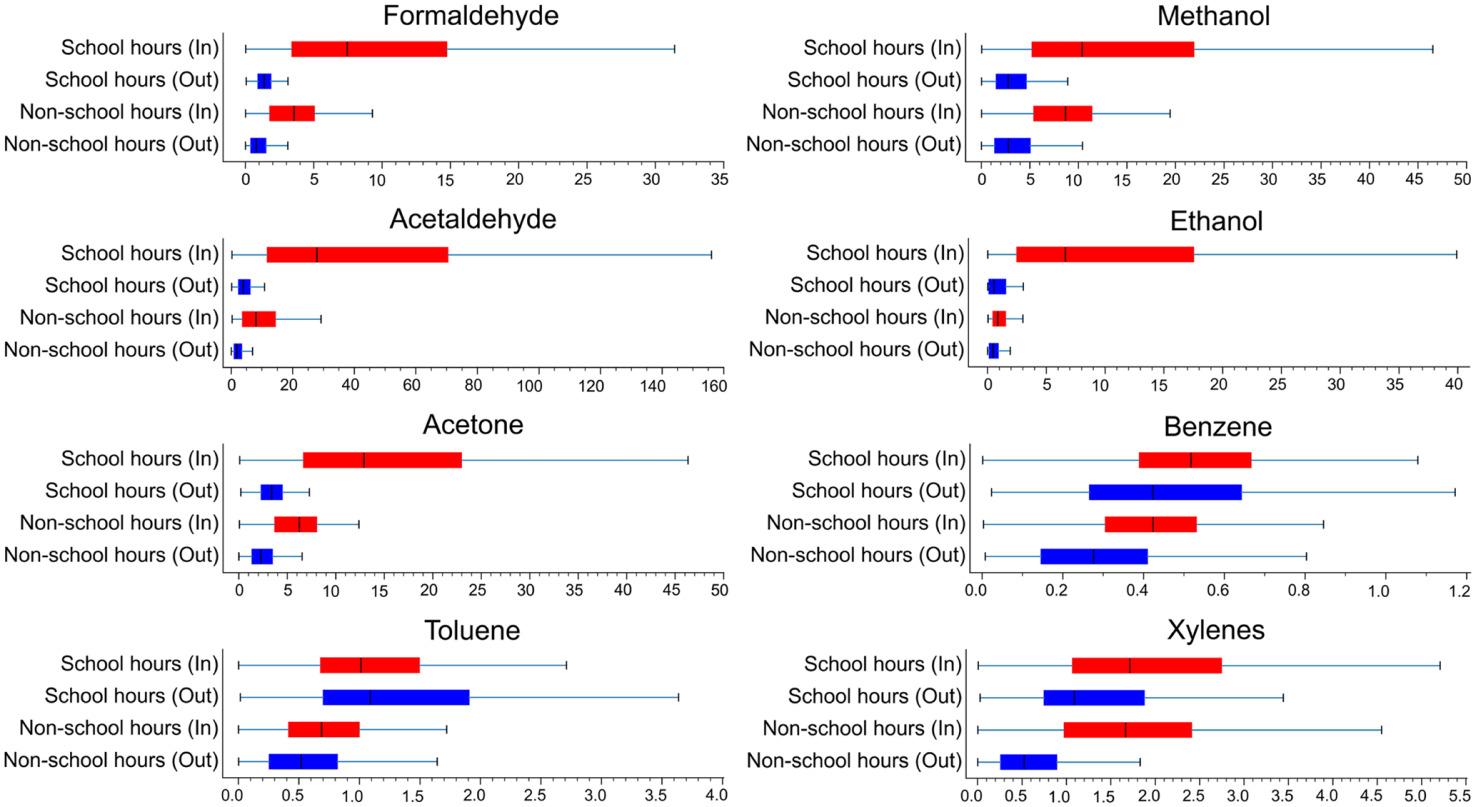
Marked variations in individual VOC levels inside (In) and outside (Out) an elementary school classroom in Athens, Greece during school or non‐school hours. The continuous measurements were performed with a Proton‐Transfer‐Reaction Mass Spectrometer during the SynAir‐G project in January 2024. Data expressed as medians, interquartile ranges and ranges (ppb, parts per billion). VOC, volatile organic compound.

Emissions from new furniture, flooring, paints, adhesives, cleaning products, personal care products, air fresheners, and ETS are common sources of VOCs [31, 42, 43]. Many different VOCs, like formaldehyde, acetaldehyde, methanol, ethanol, acetone, benzene, toluene, and xylenes, were detected in school air (Figure 5). Other relevant VOCs include vinyl chloride, isoprene, naphthalene, styrene, trimethylbenzene, phenol, dichlorobenzenes, and monoterpenes. While the composition of VOCs in indoor environments may vary depending on for example, products used and the ventilation rates, overall chronic exposure is inevitable.

Many studies have shown the health impacts of VOCs. At the EU level, the lowest concentration of interest (EU‐LCI) is a health‐based guidance value to assess VOC emissions. Of more than 90 substances assessed de novo, 40% of the EU‐LCIs are based on effects in the respiratory tract, 11% on reproductive toxicity and 37% on other types of systemic toxicity, often drawing on animal data [44].

Despite a large and growing body of research, for many VOCs data are missing, and robust data on exposures in indoor environments are lacking. Future research should standardize VOC measurement and further explore the long‐term health effects of chronic exposure to them [43, 45].

### 2.4 Semi‐Volatile Organic Compounds

Semi‐volatile organic compounds (SVOCs) are less volatile than VOCs. They are present in indoor air as vapors but are also present in the particulate phase and can be absorbed onto surfaces [46]. SVOCs include phthalates, PAHs, nicotine, etc. The exposure to SVOCs is via air inhalation, dust ingestion, dermal and oral exposure. Health effects associated with SVOCs are difficult to link to specific compounds, as the impacts of concern are chronic, sub‐lethal, and complicated by the presence of complex mixtures of SVOCs and the many routes of exposure in indoor environments. As a result of this challenge, few health‐based guidelines exist for SVOCs in indoor non‐occupational environments.

The diversity of SVOC chemical families and their properties, and the dynamic nature of indoor airflows make comprehensive characterization challenging. Characterization of chemicals in house dust has identified 2350 different compounds [47]. Certain categories of SVOCs have received focused attention in indoor environments, in particular plasticizers such as phthalate esters, bisphenols, flame retardants, polychlorinated biphenyls (PCBs), per‐ and polyfluoroalkyl substances (PFAS), PAHs, fragrance compounds and other personal care/cleaning product additives [48].

Certain SVOCs, including PCBs and PFAS, are known to bioaccumulate in humans and pose significant health risks [49, 50]. However, the absence of large cohort studies has hindered the establishment of clear links between exposure and adverse health effects. Other SVOCs, including flame retardants, have been associated with male reproductive effects, respiratory impacts on children, and neurological development [51, 52]. High PAH levels, particularly from coal use in indoor spaces, have been strongly associated with lung cancer [53]. Phthalates are widely used in consumer products and materials and are ubiquitous in indoor environments. Early‐life exposure to phthalates has been linked to an increased risk of developing childhood asthma and allergies [54]. Similarly, bisphenol A (BPA), commonly used in a wide range of plastics, is a well‐known endocrine disruptor with potential impacts on metabolic health [55].

Addressing such possible additive or synergistic effects from SVOC mixtures indoors remains a significant challenge in the field [56]. However, recent advancements in analytical technologies, data processing tools, and the increasing prevalence of large cohort studies offer promising avenues for addressing these complexities in the near future.

### 2.5 Bioaerosols

Bioaerosols, composed of microorganisms, their metabolic products, cell debris, pollen and spores, range in size within the diameter of 100 μm, with health risk increasing when a significant proportion falls within the respirable PM_2.5_ (diameter no greater than 2.5 μm) fractions [57, 58, 59, 60]. Airborne microorganisms are fundamental to both ecological balance and human health, contributing significantly to ecosystem stability and immune system development [61]. The ability of bioaerosols to travel over large distances due to evolved protective mechanisms underscores their significance in public health and environmental systems [57, 62, 63, 64, 65].

Air harbors a high diversity and abundance of human pathogens including bacteria, fungi, viruses, pollen, and insects [66, 67]. Young children, the elderly and allergic or immunocompromised individuals often suffer most, when concentrations of certain microbial species, allergens, mycotoxins and endotoxins become abnormally high [66, 68, 69, 70, 71, 72]. Aeroallergens (pollen, fungi) particularly affect sensitized individuals that is, those who have developed allergen‐specific IgE, mediating immediate‐type allergic diseases (e.g., rhinoconjunctivitis, asthma) [73].

**FIGURE 6:**
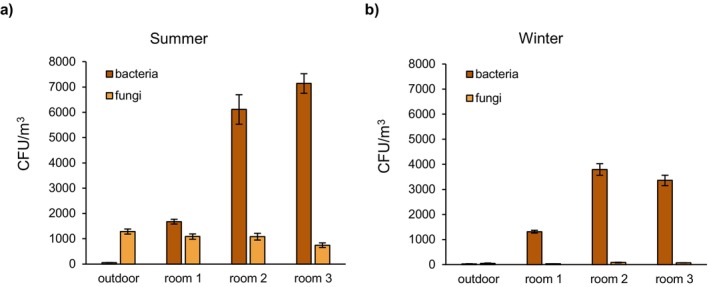
Abundance of bacteria and fungi in summer (a) and winter (b) outdoors and in classrooms of a Polish school as part of the K‐HEALTHinAIR project. Marked variations are seen between classrooms and according to the seasons. Samples (10 replicates) were collected using Mas 100 Eco impactors (Merck Millipore) during lessons in a well‐ventilated classroom (open windows, classroom 1) and in classrooms with closed windows (classrooms 2 and 3). Error bars represent standard errors of the means. Bacterial and fungal colonies were cultured on tryptone soya agar and malt extract agar at 37°C and 20°C for 2 and 5 days, respectively.

The indoor bacterial microbiome is a combination of species originating from inhabitants and outdoor air, with Gram‐positive genera dominating, such as *Micrococcus*, *Staphylococcus*, *Streptococcus*, *Kocuria*, *Corynebacterium*, *Actinobacteria*, *Arthrobacter* and *Bacillus*. Less numerous Gram‐negative bacteria, including *Enterobacter, Pseudomonas, Alcaligenes, Acinetobacter*, *Moraxella* and *Pantoea*, are of increasing concern due to the release of endotoxins [66, 68, 74, 75, 76]. The fungal microbiome in buildings without dampness problems is largely outdoor‐derived and shaped by geography, with common genera including *Cladosporium, Alternaria, Aspergillus, Trichoderma, Fusarium*, *Penicillium, Rhizopus* and *Stachybotrys* [66, 74, 75, 76]. Seasonal variations (higher in warm seasons) occur in naturally ventilated spaces, as shown in Figure 6. High humidity and insufficient ventilation alter the indoor air microbiome [68, 77], promoting fungi like biocide‐tolerant and mycoparasitic *Trichoderma* [77] and *Aspergillus*, a WHO's 2022 fungal priority pathogen [78]. Each indoor setting harbors a unique microbiome fingerprint, with bacterial communities shaped by human occupancy, whereas fungi are primarily derived from the outdoor environment [27, 66, 68, 74, 75, 76, 79, 80]. Pollen grains/spores can be transported indoors with open doors/windows, ventilation ducts and residents, and the indoor concentrations of allergens can remain elevated for a long time [81, 82, 83, 84, 85].

Opportunistic pathogens occur in lower abundance in residential spaces compared to higher‐risk environments like hospitals [86, 87, 88]. Schools are reported as buildings with the highest bioaerosol levels due to high occupancy and insufficient ventilation [74, 77, 89, 90]. Research on SARS‐CoV‐2 and the COVID‐19 pandemic has heightened attention to bioaerosols and factors shaping the structure of indoor air microbiomes and the transmission of harmful microorganisms [91, 92, 93, 94]. In poorly ventilated, crowded spaces, higher viral loads increase the risk of airborne transmission [95]. This highlights the importance of designing healthier indoor spaces to mitigate the risks associated with airborne pathogens [96, 97].

Non‐pathogenic microorganisms originating from the environment may act as benign stimuli, promoting immune resilience and reducing susceptibility to infections and allergies [98, 99, 100, 101, 102, 103, 104]. The emerging biodiversity hypothesis is based on the concept that contact with natural environments enriches the human microbiome, promotes immune balance and protects from allergy and inflammatory disorders [2]. Our immune system is protected by microbiota of the gut, skin and airways which are shaped by the biodiversity (by definition, the variability of living organisms from all sources) of our environment. Our microbiota is based on all we eat, drink, touch or inhale. Development and maintenance of mucosal tolerance are dependent on a healthy epithelial barrier as well as environmental exposure to diverse bioparticles and microbiota [2, 105]. Efficient interaction between Toll‐like receptors and the ligands of microbes and bioparticles enhances normal mucosal function and prevents allergen‐specific type 2 inflammatory events [106, 107, 108].

### 2.6 Relationship Between Outdoor and Indoor Air Quality

Outdoor air pollutants significantly impact IAQ, primarily through infiltration processes and occupant behaviors. While people spend approximately 90% of their time indoors, understanding how outdoor pollutants affect IAQ is crucial for public health [1, 38, 109]. Outdoor air pollutants can enter indoor spaces through various pathways, including open windows, doors, and ventilation systems. This infiltration is influenced by factors such as building infrastructure and weather conditions. For instance, during wildfire seasons, PM_2.5_ concentrations indoors can be more than twice the concentrations observed outdoors [110]. Homes located near major roadways have elevated levels of black carbon, CO, and NO_2_, which can cause health risks for residents [111, 112].

The characteristics of a building, such as its ventilation system, building and furnishing materials, and proximity to pollution sources, play a critical role in determining IAQ. Buildings with open windows have increased indoor black carbon and PAH levels [111]. However, buildings with mechanical ventilation systems may have higher indoor/outdoor (I/O) ratios of pollutants compared to those without, indicating that the design and operation of these systems can either mitigate or increase pollutant levels [111]. The I/O ratios of bioaerosol concentration are employed to quantify the effect of outdoor sources on indoor air microbiota [27, 70, 79, 89, 113, 114, 115, 116]. The I/O ratio is usually > 1 for bacterial concentrations, indicating that the dominant sources are occupants and their activities. In contrast, the fungal I/O ratio is usually < 1 indicating that outdoor air is a major source of the fungal microbiome. For many VOCs and SVOCs, the concentrations are significantly higher indoors than outdoors (I/O > 1) and ventilation is an important measure to reduce exposure.

In summary, outdoor air pollutants can severely compromise IAQ. The location and characteristics of buildings significantly influence this impact. Climate change, air pollution, socio‐economic factors, and urban lifestyle synergistically impact IAQ. Addressing these issues is essential for protecting public health, particularly in urban areas where pollution levels are typically high.

## 3 Relation to Health

### 3.1 Mechanisms to Affect Human Health

**FIGURE 7:**
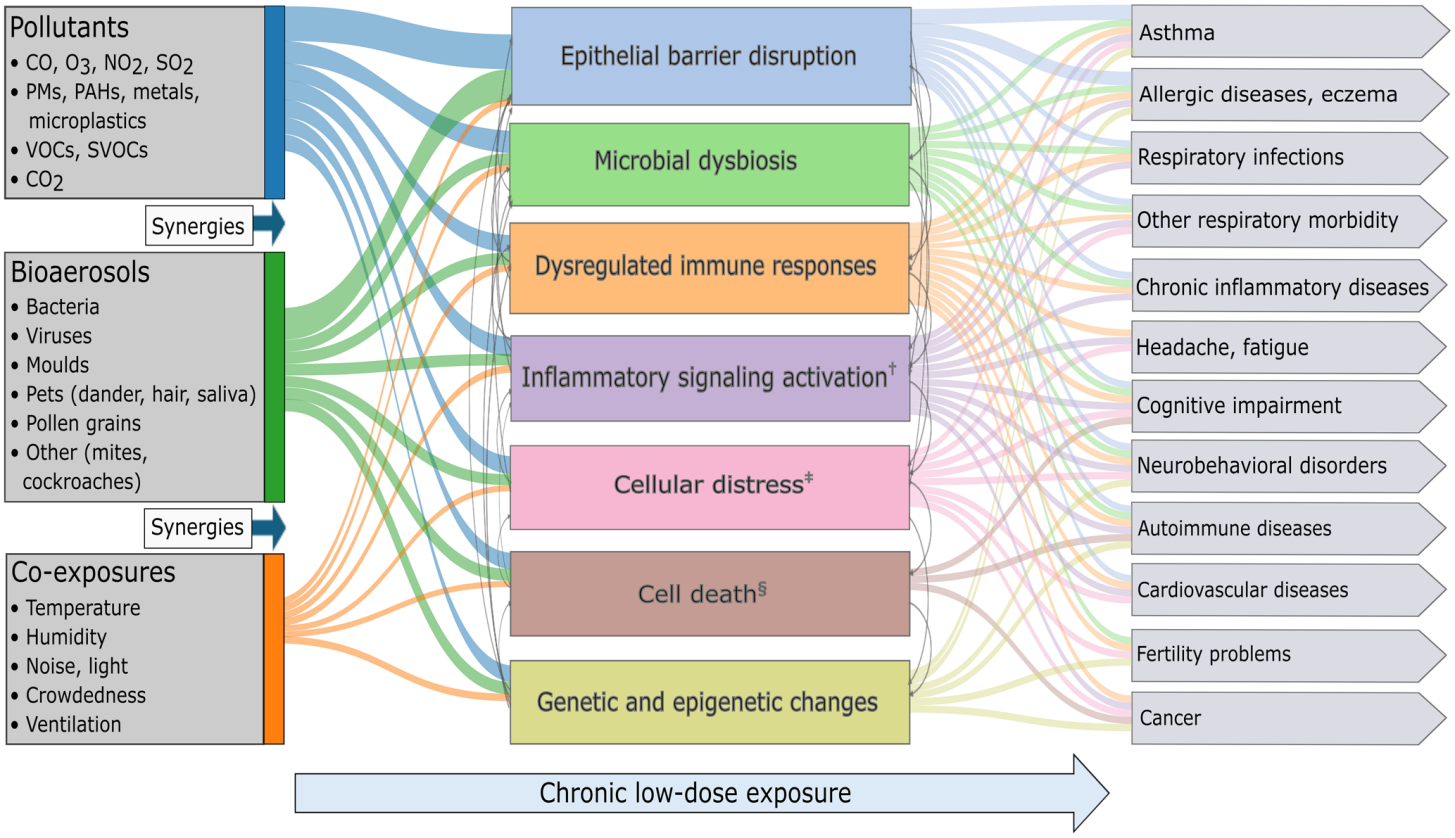
Summarization of the most important indoor exposures, mechanisms of the pathophysiological processes related to the exposure and the most significant health effects, emphasizing multi‐pollutant synergies and chronic low‐dose exposure. The complex interactions between exposure, mechanisms and health outcomes are crucial, and most health effects occur through multiple mechanisms. Some of the mechanisms are strongly linked to specific health outcomes as core mechanisms (lines) and other mechanisms are indirectly involved as contributing or susceptibility factors. ^†^Activation of inflammatory signaling pathways (MAPK, NF‐κB, AP‐1), inflammatory cytokine release, migration of activated inflammatory cells. ^‡^Cellular distress includes oxidative stress, endoplasmic reticulum stress, translation abnormalities, DNA damage and repair machinery malfunctions. ^§^Cell death includes apoptosis, necrosis, pyroptosis, autophagy, and ferroptosis. AP‐1, activator protein 1; CO, carbon monoxide; CO_2_, carbon dioxide; MAPK, mitogen‐activated protein kinase; NF‐κB, nuclear factor‐κB; NO_2_, nitrogen dioxide; O_3_, ozone; PAH, polycyclic aromatic hydrocarbon; PM, particulate matter; PM_2.5_, particulate matter with aerodynamic diameter less than or equal to 2.5 μm; SO_2_, sulfur dioxide; SVOC, semi‐volatile organic compound; VOC, volatile organic compound.

Air pollutants are known to cause various types of cell death (apoptosis, necrosis etc.), oxidative stress on cells and the endoplasmic reticulum, translation abnormalities and DNA repair machinery malfunctions, activation of inflammatory signaling pathways, and inflammatory cytokine release [117, 118]. Certain pollutants may cause DNA damage, including DNA single‐strand breaks (SSBs) and double‐strand breaks (DSBs), DNA adducts, as well as oxidative stress which can cause additional damage to proteins and lipids [119, 120, 121]. PM is thought to generate reactive oxygen species (ROS), which induce inflammation. Activated inflammatory cells may further amplify ROS generation and oxidative DNA damage [122, 123, 124]. Persistent DNA damage can compromise genome stability, increasing the risk of mutations and chromosomal abnormalities. This instability may contribute to the development of various health conditions, developmental defects and cancer, which are usually manifested with a delay of several years or even decades [125]. In addition, air pollution impairs the immune system's ability to regulate inflammation, subsequently leading to adverse health outcomes [126, 127]. Many pollutants, like SVOCs, in indoor air have been associated with effects on fertility and cardiovascular diseases [1, 51, 109, 128]. Several pathophysiological processes related to exposure to pollutants are summarized in Figure 7, which also shows that most health effects occur through multiple mechanisms. Research in this field continues to explore the specific ways indoor air pollutants affect human health, emphasizing the importance of reducing exposure [129].

Methods such as the comet and micronucleus assays are valuable tools in human biomonitoring and measuring DNA and chromosomal damage [130, 131, 132, 133, 134, 135, 136, 137, 138, 139, 140, 141, 142]. The application of these assays in studies on indoor air pollution will increase understanding of the genotoxic effects of pollutants at a cellular level and, in turn, shed light on the potential onset of many diseases, including cancer [143, 144].

### 3.2 Poor Indoor Air Quality Is Linked to Respiratory Infections

Studies in the mid‐1980s suggested for the first time that the risk of pneumonia in children is associated with IAQ at home [145]. A growing number of studies have provided evidence of a strong association between indoor air pollution and the risk of respiratory infections. In 2020, a meta‐analysis showed that household air pollution increases the risk of acute respiratory infections in both children and adults (relative risk 1.5) [128]. It showed a beneficial impact of using markedly improved cookstoves compared to traditional stoves on the risk of acute respiratory infections. Recently, a meta‐analysis showed that early life exposure to residential mold and dampness indoors increases the risk of respiratory infections in children (odds ratios [OR] 1.3–1.8) [146]. Similar to the results reported by Groot et al. [146], repairing mold‐damaged houses and offices markedly decreased respiratory infections [147].

The school environment plays a crucial role in the spread of respiratory infections, but there are only a few studies on the associations between IAQ at schools and respiratory infections. Exposure to chemical indoor air pollutants, such as formaldehyde, ethylbenzene and para‐dichlorobenzene, may increase the risk of acute respiratory infections and flu‐like symptoms (OR 1.5–2.5) [148, 149]. Studies have also shown a positive trend between high relative humidity and respiratory infections requiring antibiotic treatment [150]. Studies on school building characteristics, such as water damage and signs of moisture damage and mold odor, have shown mixed results on the occurrence of respiratory infections [151, 152, 153, 154, 155]. However, there is weak evidence that repairing mold/dampness‐damaged schools decreases the number of pupils' visits to physicians due to respiratory infections [147]. The occurrence of respiratory infections and school absenteeism due to respiratory infections has also been associated with poor ventilation in indoor settings [156, 157].

### 3.3 Indoor Air Quality and Asthma and Allergy Symptoms

Asthma is a long‐term inflammatory disease of the lower airways associated with bronchial hyperreactivity. Rapid urbanization, antibiotic usage, pollution, and climate change promote the loss of biodiversity and the onset of chronic non‐communicable illnesses such as asthma and allergies [2, 105, 158, 159]. Traffic‐related air pollution (PM_2.5_, CO, O_3_, NO_2_, and SO_2_) impairs lung growth and function, and may promote the development of asthma and allergic diseases by several mechanisms, including oxidative stress, altered barrier integrity, and induction of inflammation [18, 20, 21, 160, 161, 162]. Exposure to VOCs is also associated with a higher risk of developing asthma in young children [42, 163]. The complex interplay between environmental exposures (e.g., animal, pollen, mold allergens, viruses, tobacco smoke and air pollution) and the host shapes the risk of asthma and allergic disease development [160, 164, 165].

Childhood asthma is often associated with other allergic diseases, such as atopic eczema and allergic rhinitis. Airway inflammation often starts in childhood, when environmental stimuli such as viral respiratory tract infections, exposure to parental smoking, and NO_2_ and other airborne pollutants or allergens activate airway epithelial cells to produce type 2 inflammatory cytokines including IL‐25, IL‐33, or thymic stromal lymphopoietin [166]. This initiates a cascade that leads to chronic airway inflammation and the development of childhood asthma. Epigenetics, such as DNA methylation, is one mechanism by which environmental factors can affect gene regulation and may explain the long‐term programming of disease from early life exposures and changes in disease status over time [167, 168, 169].

Respiratory viral infections (linked to poor IAQ and human density) are the most important triggers of asthma exacerbations in children [170]. Rhinovirus (RV) infections are the main triggers and susceptibility to their infections increases with damaged airway epithelium [171]. In addition to exposure, individual susceptibility such as atopy and risk genes plays a major role [172]. For example, RV triggered early wheezing episodes are closely linked with subsequent asthma (OR up to 45 depending on cofactors such as aeroallergen sensitization or expression of 17q21 asthma risk alleles) [173, 174, 175]. Respiratory syncytial virus (RSV) bronchiolitis is associated with later non‐atopic asthma [170]. Exposure to air pollutants, such as PM and NO_2_, may also increase the risk of pediatric hospitalization due to RSV infections [176]. Respiratory infections also spread easily within close contact [177].

Exposure to multiple pollutants, viruses and pollens have multi‐synergistic effects on health. For instance, ozone, CO_2_ and NO_2_ increase pollen concentrations, which all disrupt the epithelial barrier to make it more vulnerable to viral infections [178, 179, 180, 181, 182]. Improved air quality can reduce asthma and allergy development and asthma exacerbations by reducing pollutants damaging the airway epithelium, allergens promoting chronic airway inflammation and infectious agents triggering respiratory attacks, and increasing biodiversity in the air and surfaces to strengthen the immune system [2, 4, 183]. A better understanding of complex interactions between environmental factors and biological processes is crucial in developing new strategies for the prevention of asthma and allergic diseases.

### 3.4 Respiratory Health and Other Morbidity

The accumulation of PM and PAHs over time makes chronic exposure inevitable. Long‐term exposure to these pollutants is associated with an increased risk of cancer [184, 185], impaired lung function [186] and can lead to respiratory health outcomes such as asthma and chronic obstructive pulmonary disease (COPD) [117, 187]. Even healthy people exposed to elevated concentrations of PM and PAHs may develop respiratory symptoms [188]. Higher PAH levels also increase proinflammatory serum cytokines [188]. Studies indicate that short‐term PM exposure also increases respiratory problems, coughing and reduced lung function [45], enhancing the risk of hospital admissions due to asthma [189] and COPD [190] exacerbations. Especially PM_2.5_ is small enough to reach the alveoli [16, 18, 30, 191], where it may cause inflammation and oxidative stress, and consequently, worsen respiratory conditions and increase cardiovascular risks [19].

Exposures to VOCs have been found to increase wheezing, coughing, and reduce lung function [45, 192]. Children exposed to VOCs in daycares, schools, and homes are facing higher risks of asthma and other respiratory conditions [42]. Elderly individuals in long‐term care facilities have been found to experience worsened respiratory conditions when exposed to high VOC levels [193]. For individuals with asthma, exposure to specific VOCs, such as aromatic and aliphatic compounds, worsens symptoms [45]. Exposure to limonene, a common fragrance, was shown to increase the risk of new‐onset asthma [45]. Moreover, poor IAQ is linked to cardiovascular diseases, cancer, cognitive and fertility problems, and reduced life expectancy [51, 53, 109, 111, 128].

### 3.5 Indoor Air Quality Affects Cognitive Function and Developing Brains

Elevated CO_2_ concentrations (> 1000 ppm) can cause symptoms such as headaches, dizziness, fatigue, and impaired cognitive function [22]. Prolonged exposure to very high concentrations (> 5000 ppm) may lead to more severe effects, including shortness of breath and increased heart rate [23]. CO binds to hemoglobin and impairs oxygen delivery, causing headaches, dizziness, and even coma or death [16, 19, 20, 21, 194]. Chronic exposure, even at low levels, also increases cardiovascular risks and hospitalizations, particularly in the elderly [16].

IAQ has been increasingly linked to cognitive and behavioral development outcomes, particularly in children, who are more vulnerable to environmental factors due to their developing brains. Poor IAQ, characterized by high levels of pollutants such as, PM, VOCs, SVOCs, CO_2_ and NO_2_, is associated with lower cognitive performance, reduced memory, shorter attention span, and slower cognitive processing, particularly in children [195, 196]. Chronic exposure to indoor air pollutants may affect language development and intelligence quotient scores [197].

Pollutants like ultrafine particles can cross the blood–brain barrier, leading to neuroinflammation and oxidative stress, which are implicated in neurodevelopmental delays and disorders in children [198]. Poor IAQ is also connected to behavioral issues such as attention‐deficit/hyperactivity disorder (ADHD), anxiety, and social interaction problems [199]. Prenatal and early life exposure to higher levels of indoor air pollutants may predispose children to behavioral disturbances, including impulsivity and emotional dysregulation [200].

Adequate ventilation plays a crucial role in improving IAQ, which in turn can mitigate some of these adverse cognitive and behavioral developmental effects. Improved ventilation, air filtration systems, and reducing sources of indoor air pollutants (like tobacco smoke and chemical cleaners) are essential interventions and can be regulated with healthy public policy‐making. Maintaining high IAQ is vital for supporting healthy cognitive and behavioral development, especially in young children, where environmental exposure can have long‐term consequences.

## 4 Summary and Gaps for Further Research

Several studies have shown an association between poor IAQ and physical, mental as well as immunological health problems. PMs, PAHs, CO, NO_2_, and O_3_ from transportation, industry, smoking and indoor heating stoves significantly worsen IAQ and cause inflammation, oxidative stress, cardiovascular and cognitive problems, cancer and exacerbate asthma and COPD [14, 18, 19, 20, 160]. Indoor VOCs and SVOCs from furniture, flooring, paints, adhesives, cleaning products and other chemicals pose risks for respiratory, cognitive and reproductive health [44]. Bioaerosols of bacteria, viruses and molds shape immune system development [61]. While pathogenic microorganisms cause infections and exacerbate allergies, non‐pathogenic microorganisms act as benign stimuli and enhance resilience [98, 99, 100, 101, 102, 103, 104]. Poor IAQ can negatively affect health in general and increase absenteeism [60, 68, 77, 90, 195, 196]. Even at “acceptable” levels, chronic low‐dose exposure to indoor air pollutants may impair epithelial integrity, microbiota balance, inflammatory regulation, and long‐term human health.

The IDEAL cluster identified several gaps for future research:

1. Complex interactions between environmental factors and dynamic biological processes are not sufficiently understood, requiring further mechanistic and multi‐disciplinary research.
2. Robust data for toxicity and exposure‐response relationships, in particular for long‐term health effects are missing.
3. Standardization of IAQ measurements is vital, both for comparability across primary studies and for implementation of IAQ guidelines.
4. Assays detecting DNA damage and genome instability caused by indoor air pollutants will be useful for detecting genotoxic effects on a cellular level.
5. Tailoring IAQ, by air purifiers, green walls or other biomaterials, to strengthen the immune system, needs more research.
6. A better overview of the actual hazardous chemical and biological determinants in indoor environments is needed.
7. Understanding multi‐pollutant synergies is crucial for improving IAQ, as combined pollutant effects can be more harmful than individual exposures and require integrated mitigation strategies.

The data on the effects and mechanisms of IAQ on health is limited and this was the reason for launching the IDEAL cluster projects, which aim to promote knowledge of all the aforementioned gaps. IAQ is a matter of public concern. Effective prevention and management of indoor air exposure involve source control, ventilation, and regular monitoring of pollutant levels. Strategies like improved ventilation, air purification, low‐emission materials, and public education are essential for protecting public health and need to be based on scientific research. Recent advances in analytical technologies and data processing, and the increasing prevalence of large cohort studies offer promising tools for addressing these complex questions. Public awareness is crucial in reducing pollutant concentrations linked to everyday products and activities and improving legislation.

## Author Contributions

Study conception and design: All authors. Critical revision of the manuscript: All authors.

## Funding

This work was supported by Next Generation EU; European Regional Development Fund; Croatian Science Foundation; UK Research and Innovation; National Health and Medical Research Council; UK Research and Innovation; State Secretariat for Education, Research and Innovation; Horizon Europe.

## Conflicts of Interest

The authors declare no conflicts of interest.

## Data Availability

The data that support the findings of this study are available from the corresponding author upon reasonable request.

## References

[1] P. L. Jenkins , T. J. Phillips , E. J. Mulberg , and S. P. Hui , “Activity Patterns of Californians: Use of and Proximity to Indoor Pollutant Sources,” Atmospheric Environment Part A General Topics 26, no. 12 (1992): 2141–2148.

[2] T. Haahtela , “A Biodiversity Hypothesis,” Allergy 74, no. 8 (2019): 1445–1456. 30835837 10.1111/all.13763

[3] J. Moya , C. F. Bearer , and R. A. Etzel , “Children's Behavior and Physiology and How It Affects Exposure to Environmental Contaminants,” Pediatrics 113, no. Supplement_3 (2004): 996–1006. 15060192

[4] B. Brunekreef and S. T. Holgate , “Air Pollution and Health,” Lancet 360, no. 9341 (2002): 1233–1242. 12401268 10.1016/S0140-6736(02)11274-8

[5] T. V. Van , D. Park , and Y. C. Lee , “Indoor Air Pollution, Related Human Diseases, and Recent Trends in the Control and Improvement of Indoor Air Quality,” International Journal of Environmental Research and Public Health 17, no. 8 (2020): 2927. 32340311 10.3390/ijerph17082927 PMC7215772

[6] J. Samet , F. Holguin , and M. Buran , “The Health Effects of Indoor Air Pollution,” in Handbook of Indoor Air Quality, ed. Y. Zhang , P. K. Hopke , and C. Mandin (Springer Nature Singapore, 2022), 1141–1187.

[7] Z. Wang , G. W. Walker , D. C. G. Muir , and K. Nagatani‐Yoshida , “Toward a Global Understanding of Chemical Pollution: A First Comprehensive Analysis of National and Regional Chemical Inventories,” Environmental Science & Technology 54, no. 5 (2020): 2575–2584. 31968937 10.1021/acs.est.9b06379

[8] C. Sonne , C. Xia , P. Dadvand , A. C. Targino , and S. S. Lam , “Indoor Volatile and Semi‐Volatile Organic Toxic Compounds: Need for Global Action,” Journal of Building Engineering 62 (2022): 105344.

[9] EEA , “Air Pollution,” (2024), https://www.eea.europa.eu/en/topics/in‐depth/air‐pollution.

[10] EEA , “Harm to Human Health From Air Pollution in Europe: Burden of Disease 2023,” (2023), https://www.eea.europa.eu/publications/harm‐to‐human‐health‐from‐air‐pollution.

[11] C. E. Delft , Health Costs of Air Pollution in European Cities and the Linkage With Transport (Publication Code 20.190272.134) (CE Delft, 2020).

[12] W. Y. Liu , Z. L. Jiesisibieke , and T. H. Tung , “Effect of Asthma Education on Health Outcomes in Children: A Systematic Review,” Archives of Disease in Childhood 107, no. 12 (2022): 1100. 35197244 10.1136/archdischild-2021-323496 PMC9685736

[13] A. A. Arif and S. M. Shah , “Association Between Personal Exposure to Volatile Organic Compounds and Asthma Among US Adult Population,” International Archives of Occupational and Environmental Health 80, no. 8 (2007): 711–719. 17357796 10.1007/s00420-007-0183-2

[14] World Health Organization Regional Office for Europe , WHO Guidelines for Indoor Air Quality: Selected Pollutants (WHO, 2010). 23741784

[15] E. J. Sugeng , M. de Cock , P. E. G. Leonards , and M. van de Bor , “Toddler Behavior, the Home Environment, and Flame Retardant Exposure,” Chemosphere 252 (2020): 126588. 32229360 10.1016/j.chemosphere.2020.126588

[16] S. Vardoulakis , E. Giagloglou , S. Steinle , et al., “Indoor Exposure to Selected Air Pollutants in the Home Environment: A Systematic Review,” International Journal of Environmental Research and Public Health 17, no. 23 (2020): 8972. 33276576 10.3390/ijerph17238972 PMC7729884

[17] A. Goshua , C. A. Akdis , and K. C. Nadeau , “World Health Organization Global Air Quality Guideline Recommendations: Executive Summary,” Allergy 77, no. 7 (2022): 1955–1960. 35060140 10.1111/all.15224 PMC12052406

[18] P. Kumar , A. B. Singh , T. Arora , S. Singh , and R. Singh , “Critical Review on Emerging Health Effects Associated With the Indoor Air Quality and Its Sustainable Management,” Science of the Total Environment 872 (2023): 162163. 36781134 10.1016/j.scitotenv.2023.162163

[19] I. Manisalidis , E. Stavropoulou , A. Stavropoulos , and E. Bezirtzoglou , “Environmental and Health Impacts of Air Pollution: A Review,” Frontiers in Public Health 8 (2020): 14. 32154200 10.3389/fpubh.2020.00014 PMC7044178

[20] J. A. Bernstein , N. Alexis , H. Bacchus , et al., “The Health Effects of Nonindustrial Indoor Air Pollution,” Journal of Allergy and Clinical Immunology 121, no. 3 (2008): 585–591. 18155285 10.1016/j.jaci.2007.10.045

[21] A. P. Jones , “Indoor Air Quality and Health,” Atmospheric Environment 33, no. 28 (1999): 4535–4564.

[22] S. Usha , M. J. Mendell , S. Krishnamurthy , et al., “Is CO_2_ an Indoor Pollutant? Direct Effects of Low‐To‐Moderate CO_2_ Concentrations on Human Decision‐Making Performance,” Environmental Health Perspectives 120, no. 12 (2012): 1671–1677. 23008272 10.1289/ehp.1104789 PMC3548274

[23] Y. Fan , X. Cao , J. Zhang , D. Lai , and L. Pang , “Short‐Term Exposure to Indoor Carbon Dioxide and Cognitive Task Performance: A Systematic Review and Meta‐Analysis,” Building and Environment 237 (2023): 110331.

[24] Y. Huang , Z. Yang , and Z. Gao , “Contributions of Indoor and Outdoor Sources to Ozone in Residential Buildings in Nanjing,” International Journal of Environmental Research and Public Health 16, no. 14 (2019): 2587. 31331082 10.3390/ijerph16142587 PMC6678623

[25] WHO , WHO Global Air Quality Guidelines: Particulate Matter (PM_2.5_ and PM_10_), Ozone, Nitrogen Dioxide, Sulfur Dioxide and Carbon Monoxide (WHO, 2021), https://www.who.int/publications/i/item/9789240034228. 34662007

[26] C. Alves , T. Nunes , J. Silva , and M. Duarte , “Comfort Parameters and Particulate Matter (PM_10_ and PM_2.5_) in School Classrooms and Outdoor Air,” Aerosol and Air Quality Research 13, no. 5 (2013): 1521–1535.

[27] A. Badyda , A. Muszyński , K. Affek , et al., “Schools and Indoor Air Quality: Seasonal Variation,” European Journal of Public Health 34, no. Supplement_3 (2024): 797.

[28] E. Dovrou , C. Kaltsonoudis , S. Androulakis , I. Apostolopoulos , A. Simonati , and S. N. Pandis , “Evaluation of Air Quality in a Primary School Classroom During Wintertime,” Indoor Air 2024, no. 1 (2024): 7888273.

[29] F. Amato , I. Rivas , M. Viana , et al., “Sources of Indoor and Outdoor PM_2.5_ Concentrations in Primary Schools,” Science of the Total Environment 490 (2014): 757–765. 24907610 10.1016/j.scitotenv.2014.05.051

[30] Z. Li , Q. Wen , and R. Zhang , “Sources, Health Effects and Control Strategies of Indoor Fine Particulate Matter (PM_2.5_): A Review,” Science of the Total Environment 586 (2017): 610–622. 28216030 10.1016/j.scitotenv.2017.02.029

[31] J. Madureira , A. Mendes , and J. P. Teixeira , “Evaluation of a Smoke‐Free Law on Indoor Air Quality and on Workers' Health in Portuguese Restaurants,” Journal of Occupational and Environmental Hygiene 11, no. 4 (2014): 201–209. 24579749 10.1080/15459624.2013.852279

[32] M. Wang , S. Jia , S. H. Lee , A. Chow , and M. Fang , “Polycyclic Aromatic Hydrocarbons (PAHs) in Indoor Environments Are Still Imposing Carcinogenic Risk,” Journal of Hazardous Materials 409 (2021): 124531. 33250308 10.1016/j.jhazmat.2020.124531

[33] L. Fappiano , E. Caracci , A. Iannone , et al., “Emission Rates of Particle‐Bound Heavy Metals and Polycyclic Aromatic Hydrocarbons in PM Fractions From Indoor Combustion Sources,” Building and Environment 265 (2024): 112033.

[34] C. M. F. Rosales , J. Jung , and M. G. Cayetano , “Emissions and Chemical Components of PM_2.5_ From Simulated Cooking Conditions Using Traditional Cookstoves and Fuels Under a Dilution Tunnel System,” Aerosol and Air Quality Research 21, no. 7 (2021): 200581.

[35] G. Drago , C. Perrino , S. Canepari , et al., “Relationship Between Domestic Smoking and Metals and Rare Earth Elements Concentration in Indoor PM_2.5_ ,” Environmental Research 165 (2018): 71–80. 29674239 10.1016/j.envres.2018.03.026

[36] P. E. Rasmussen , C. Levesque , M. Chénier , and H. D. Gardner , “Contribution of Metals in Resuspended Dust to Indoor and Personal Inhalation Exposures: Relationships Between PM_10_ and Settled Dust,” Building and Environment 143 (2018): 513–522.

[37] H. Guo , M. Li , Y. Lyu , T. Cheng , J. Xv , and X. Li , “Size‐Resolved Particle Oxidative Potential in the Office, Laboratory, and Home: Evidence for the Importance of Water‐Soluble Transition Metals,” Environmental Pollution 246 (2019): 704–709. 30623827 10.1016/j.envpol.2018.12.094

[38] A. Wierzbicka , Y. Omelekhina , A. T. Saber , et al., “Indoor PM_2.5_ From Occupied Residences in Sweden Caused Higher Inflammation in Mice Compared to Outdoor PM_2.5_ ,” Indoor Air 32, no. 12 (2022): e13177. 36567521 10.1111/ina.13177 PMC10107884

[39] S. Borowska and M. M. Brzóska , “Metals in Cosmetics: Implications for Human Health,” Journal of Applied Toxicology 35, no. 6 (2015): 551–572. 25809475 10.1002/jat.3129

[40] Y. Meng , Y. Li , N. Zheng , et al., “Potential Health Risks of Metals in Skin Care Products Used by Chinese Consumers Aged 19–29 Years,” Ecotoxicology and Environmental Safety 216 (2021): 112184. 33839485 10.1016/j.ecoenv.2021.112184

[41] S. Ardicli , O. Ardicli , D. Yazici , et al., “Epithelial Barrier Dysfunction and Associated Diseases in Companion Animals: Differences and Similarities Between Humans and Animals and Research Needs,” Allergy 79, no. 12 (2024): 3238–3268. 39417247 10.1111/all.16343 PMC11657079

[42] N. Liu , Z. Bu , W. Liu , et al., “Health Effects of Exposure to Indoor Volatile Organic Compounds From 1980 to 2017: A Systematic Review and Meta‐Analysis,” Indoor Air 32, no. 5 (2022): e13038. 35622720 10.1111/ina.13038

[43] K. L. Alford and N. Kumar , “Pulmonary Health Effects of Indoor Volatile Organic Compounds – A Meta‐Analysis,” International Journal of Environmental Research and Public Health 18, no. 4 (2021): 1578. 33562372 10.3390/ijerph18041578 PMC7914726

[44] EU‐LCU Subgroup , “EU‐LCI Values Summary Fact Sheets, Available Online: Documents and Glossary – European Commission (europa.eu),” (2023).

[45] C. A. Paterson , R. A. Sharpe , T. Taylor , and K. Morrissey , “Indoor PM_2.5_, VOCs and Asthma Outcomes: A Systematic Review in Adults and Their Home Environments,” Environmental Research 202 (2021): 111631. 34224711 10.1016/j.envres.2021.111631

[46] J. P. D. Abbatt and C. Wang , “The Atmospheric Chemistry of Indoor Environments,” Environmental Science: Processes & Impacts 22, no. 1 (2020): 25–48. 31712796 10.1039/c9em00386j

[47] P. Rostkowski , P. Haglund , R. Aalizadeh , et al., “The Strength in Numbers: Comprehensive Characterization of House Dust Using Complementary Mass Spectrometric Techniques,” Analytical and Bioanalytical Chemistry 411, no. 10 (2019): 1957–1977. 30830245 10.1007/s00216-019-01615-6 PMC6458998

[48] L. Lucattini , G. Poma , A. Covaci , J. de Boer , M. H. Lamoree , and P. E. G. Leonards , “A Review of Semi‐Volatile Organic Compounds (SVOCs) in the Indoor Environment: Occurrence in Consumer Products, Indoor Air and Dust,” Chemosphere 201 (2018): 466–482. 29529574 10.1016/j.chemosphere.2018.02.161

[49] C. M. A. Eichler and J. C. Little , “A Framework to Model Exposure to Per‐ and Polyfluoroalkyl Substances in Indoor Environments,” Environmental Science: Processes & Impacts 22, no. 3 (2020): 500–511. 32141451 10.1039/c9em00556k

[50] H. V. Andersen , B. Kolarik , N. S. Nielsen , et al., “Indoor Air Concentrations of PCB in a Contaminated Building Estate and Factors of Importance for the Variance,” Building and Environment 204 (2021): 108135.

[51] J. D. Meeker and H. M. Stapleton , “House Dust Concentrations of Organophosphate Flame Retardants in Relation to Hormone Levels and Semen Quality Parameters,” Environmental Health Perspectives 118, no. 3 (2010): 318–323. 20194068 10.1289/ehp.0901332 PMC2854757

[52] A. Mendy , Z. Percy , J. M. Braun , et al., “Exposure to Dust Organophosphate and Replacement Brominated Flame Retardants During Infancy and Risk of Subsequent Adverse Respiratory Outcomes,” Environmental Research 235 (2023): 116560. 37419195 10.1016/j.envres.2023.116560 PMC10528780

[53] Q. Lan , X. He , M. Shen , et al., “Variation in Lung Cancer Risk by Smoky Coal Subtype in Xuanwei, China,” International Journal of Cancer 123, no. 9 (2008): 2164–2169. 18712724 10.1002/ijc.23748 PMC2974309

[54] G. Navaranjan , M. L. Diamond , S. A. Harris , et al., “Early Life Exposure to Phthalates and the Development of Childhood Asthma Among Canadian Children,” Environmental Research 197 (2021): 110981. 33691158 10.1016/j.envres.2021.110981

[55] M. Liu , S. Jia , T. Dong , et al., “The Occurrence of Bisphenol Plasticizers in Paired Dust and Urine Samples and Its Association With Oxidative Stress,” Chemosphere 216 (2019): 472–478. 30388685 10.1016/j.chemosphere.2018.10.090

[56] L. Zhu , P. Hajeb , P. Fauser , and K. Vorkamp , “Endocrine Disrupting Chemicals in Indoor Dust: A Review of Temporal and Spatial Trends, and Human Exposure,” Science of the Total Environment 874 (2023): 162374. 36828075 10.1016/j.scitotenv.2023.162374

[57] A. C. Tastassa , Y. Sharaby , and N. Lang‐Yona , “Aeromicrobiology: A Global Review of the Cycling and Relationships of Bioaerosols With the Atmosphere,” Science of the Total Environment 912 (2024): 168478. 37967625 10.1016/j.scitotenv.2023.168478

[58] T. Y. Poh , N. A. B. M. Ali , M. Mac Aogáin , et al., “Inhaled Nanomaterials and the Respiratory Microbiome: Clinical, Immunological and Toxicological Perspectives,” Particle and Fibre Toxicology 15, no. 1 (2018): 46. 30458822 10.1186/s12989-018-0282-0 PMC6245551

[59] J. S. Bin , H. S. Ko , K. J. Heo , J. H. Shin , and J. H. Jung , “Size Distribution and Concentration of Indoor Culturable Bacterial and Fungal Bioaerosols,” Atmospheric Environment: X 15 (2022): 100182.

[60] E. Brągoszewska and A. Mainka , “Assessment of Personal Deposited Dose and Particle Size Distribution of Bacterial Aerosol in Kindergarten Located in Southern Poland,” Environmental Pollution 343 (2024): 123208. 38142028 10.1016/j.envpol.2023.123208

[61] G. A. W. Rook , “The Old Friends Hypothesis: Evolution, Immunoregulation and Essential Microbial Inputs,” Frontiers in Allergy 4 (2023): 1220481. 37772259 10.3389/falgy.2023.1220481 PMC10524266

[62] R. Lappan , J. Thakar , L. Molares Moncayo , et al., “The Atmosphere: A Transport Medium or an Active Microbial Ecosystem?,” ISME Journal 18, no. 1 (2024): wrae092. 38804464 10.1093/ismejo/wrae092 PMC11214262

[63] W. Smets , S. Moretti , S. Denys , and S. Lebeer , “Airborne Bacteria in the Atmosphere: Presence, Purpose, and Potential,” Atmospheric Environment 139 (2016): 214–221.

[64] R. Tignat‐Perrier , A. Dommergue , A. Thollot , O. Magand , T. M. Vogel , and C. Larose , “Microbial Functional Signature in the Atmospheric Boundary Layer,” Biogeosciences 17, no. 23 (2020): 6081–6095.

[65] X. Rodó , S. Pozdniakova , S. Borràs , et al., “Microbial Richness and Air Chemistry in Aerosols Above the PBL Confirm 2,000‐Km Long‐Distance Transport of Potential Human Pathogens,” National Academy of Sciences of the United States of America 121, no. 38 (2024): e2404191121. 10.1073/pnas.2404191121 PMC11420185 39250672

[66] E. Carrazana , T. Ruiz‐Gil , S. Fujiyoshi , et al., “Potential Airborne Human Pathogens: A Relevant Inhabitant in Built Environments but Not Considered in Indoor Air Quality Standards,” Science of the Total Environment 901 (2023): 165879. 37517716 10.1016/j.scitotenv.2023.165879

[67] T. Li , K. Feng , S. Wang , et al., “Beyond Water and Soil: Air Emerges as a Major Reservoir of Human Pathogens,” Environment International 190 (2024): 108869. 38968831 10.1016/j.envint.2024.108869

[68] H. Chawla , P. Anand , K. Garg , et al., “A Comprehensive Review of Microbial Contamination in the Indoor Environment: Sources, Sampling, Health Risks, and Mitigation Strategies,” Frontiers in Public Health 11 (2023): 1285393. 38074709 10.3389/fpubh.2023.1285393 PMC10701447

[69] A. G. Fakunle , N. Jafta , A. Bossers , et al., “Childhood Lower Respiratory Tract Infections Linked to Residential Airborne Bacterial and Fungal Microbiota,” Environmental Research 231 (2023): 116063. 37156352 10.1016/j.envres.2023.116063

[70] A. Hassan , M. Zeeshan , and M. F. Bhatti , “Indoor and Outdoor Microbiological Air Quality in Naturally and Mechanically Ventilated University Libraries,” Atmospheric Pollution Research 12, no. 8 (2021): 101136.

[71] K. N. M. Isa , J. Jalaludin , S. M. Elias , et al., “Metagenomic Characterization of Indoor Dust Fungal Associated With Allergy and Lung Inflammation Among School Children,” Ecotoxicology and Environmental Safety 221 (2021): 112430. 34147866 10.1016/j.ecoenv.2021.112430

[72] R. A. Sharpe , N. Bearman , C. R. Thornton , K. Husk , and N. J. Osborne , “Indoor Fungal Diversity and Asthma: A Meta‐Analysis and Systematic Review of Risk Factors,” Journal of Allergy and Clinical Immunology 135, no. 1 (2015): 110–122. 25159468 10.1016/j.jaci.2014.07.002

[73] M. H. Shamji , R. Valenta , T. Jardetzky , et al., “The Role of Allergen‐Specific IgE, IgG and IgA in Allergic Disease,” Allergy 76, no. 12 (2021): 3627–3641. 33999439 10.1111/all.14908 PMC8601105

[74] R. Jabeen , M. I. Kizhisseri , S. N. Mayanaik , and M. M. Mohamed , “Bioaerosol Assessment in Indoor and Outdoor Environments: A Case Study From India,” Scientific Reports 13, no. 1 (2023): 18066. 37872255 10.1038/s41598-023-44315-z PMC10593752

[75] S. K. Shin , J. Kim , S. Ha , et al., “Metagenomic Insights Into the Bioaerosols in the Indoor and Outdoor Environments of Childcare Facilities,” PLoS One 10, no. 5 (2015): e0126960. 26020512 10.1371/journal.pone.0126960 PMC4447338

[76] T. Rejc , A. Kukec , M. Bizjak , and K. GodičTorkar , “Microbiological and Chemical Quality of Indoor Air in Kindergartens in Slovenia,” International Journal of Environmental Health Research 30, no. 1 (2020): 49–62. 30734572 10.1080/09603123.2019.1572870

[77] C. Vornanen‐Winqvist , K. Järvi , M. A. Andersson , et al., “Exposure to Indoor Air Contaminants in School Buildings With and Without Reported Indoor Air Quality Problems,” Environment International 141 (2020): 105781. 32417615 10.1016/j.envint.2020.105781

[78] N. van Rhijn , S. Arikan‐Akdagli , J. Beardsley , et al., “Beyond Bacteria: The Growing Threat of Antifungal Resistance,” Lancet 404, no. 10457 (2024): 1017–1018. 39277286 10.1016/S0140-6736(24)01695-7

[79] J. Ye , H. Qian , J. Zhang , et al., “Concentrations and Size‐Resolved I/O Ratios of Household Airborne Bacteria and Fungi in Nanjing, Southeast China,” Science of the Total Environment 774 (2021): 145559.

[80] S. Sadrizadeh , R. Yao , F. Yuan , et al., “Indoor Air Quality and Health in Schools: A Critical Review for Developing the Roadmap for the Future School Environment,” Journal of Building Engineering 57 (2022): 104908.

[81] E. Yli‐Panula , “Allergenicity of Grass Pollen in Settled Dust in Rural and Urban Homes in Finland,” Grana 36, no. 5 (1997): 306–310.

[82] I. Sauliene , A. Valiulis , I. Keriene , et al., “Airborne Pollen and Fungi Indoors: Evidence From Primary Schools in Lithuania,” Heliyon 9, no. 1 (2023): e12668. 36685406 10.1016/j.heliyon.2022.e12668 PMC9850001

[83] W. Wang , H. Kikumoto , C. Lin , W. Oh , M. Han , and R. Ooka , “Relationship Between Natural Ventilation Modes and Indoor/Outdoor Ratio of Japanese Cedar Pollen and Cry j 1 Allergen,” Building and Environment 265 (2024): 111961.

[84] J. Jantunen and K. Saarinen , “Intrusion of Airborne Pollen Through Open Windows and Doors,” Aerobiologia (Bologna) 25, no. 3 (2009): 193–201.

[85] J. Jantunen and K. Saarinen , “Pollen Transport by Clothes,” Aerobiologia (Bologna) 27, no. 4 (2011): 339–343.

[86] S. G. Tringe , T. Zhang , X. Liu , et al., “The Airborne Metagenome in an Indoor Urban Environment,” PLoS One 3, no. 4 (2008): e1862. 18382653 10.1371/journal.pone.0001862 PMC2270337

[87] A. M. Madsen , S. Moslehi‐Jenabian , M. Frankel , J. K. White , and M. W. Frederiksen , “Airborne Bacterial Species in Indoor Air and Association With Physical Factors,” UCL Open Environ 5, no. 1 (2023): e056. 37229345 10.14324/111.444/ucloe.000056 PMC10208329

[88] Y. A. Atalay , E. Mengistie , A. Tolcha , et al., “Indoor Air Bacterial Load and Antibiotic Susceptibility Pattern of Isolates at Adare General Hospital in Hawassa, Ethiopia,” Frontiers in Public Health 11 (2023): 1194850. 38026319 10.3389/fpubh.2023.1194850 PMC10653387

[89] K. Guo , H. Qian , D. Zhao , et al., “Indoor Exposure Levels of Bacteria and Fungi in Residences, Schools, and Offices in China: A Systematic Review,” Indoor Air 30, no. 6 (2020): 1147–1165. 32845998 10.1111/ina.12734

[90] P. Wargocki , J. A. Porras‐Salazar , S. Contreras‐Espinoza , and W. Bahnfleth , “The Relationships Between Classroom Air Quality and Children's Performance in School,” Building and Environment 173 (2020): 106749.

[91] L. Ciric , “Microbes in the Built Environment,” Scientific Reports 12, no. 1 (2022): 8732. 35650292 10.1038/s41598-022-12254-w PMC9160266

[92] S. W. Kembel , J. F. Meadow , T. K. O'Connor , et al., “Architectural Design Drives the Biogeography of Indoor Bacterial Communities,” PLoS One 9, no. 1 (2014): e87093. 24489843 10.1371/journal.pone.0087093 PMC3906134

[93] A. Toyoda , Y. Shibata , Y. Matsuo , et al., “Diversity and Compositional Differences of the Airborne Microbiome in a Biophilic Indoor Environment,” Scientific Reports 13, no. 1 (2023): 8179. 37210416 10.1038/s41598-023-34928-9 PMC10199911

[94] S. Rai , D. K. Singh , and A. Kumar , “Microbial, Environmental and Anthropogenic Factors Influencing the Indoor Microbiome of the Built Environment,” Journal of Basic Microbiology 61, no. 4 (2021): 267–292. 33522603 10.1002/jobm.202000575

[95] S. Tang , Y. Mao , R. M. Jones , et al., “Aerosol Transmission of SARS‐CoV‐2? Evidence, Prevention and Control,” Environment International 144 (2020): 106039. 32822927 10.1016/j.envint.2020.106039 PMC7413047

[96] P. Piscitelli , A. Miani , L. Setti , et al., “The Role of Outdoor and Indoor Air Quality in the Spread of SARS‐CoV‐2: Overview and Recommendations by the Research Group on COVID‐19 and Particulate Matter (RESCOP Commission),” Environmental Research 211 (2022): 113038. 35231456 10.1016/j.envres.2022.113038 PMC8881809

[97] National Academies of Sciences, Engineering, and Medicine , Microbiomes of the Built Environment: A Research Agenda for Indoor Microbiology, Human Health, and Buildings (National Academies Press, 2017). 29035489

[98] J. M. Robinson and M. F. Breed , “The Aerobiome‐Health Axis: A Paradigm Shift in Bioaerosol Thinking,” Trends in Microbiology 31, no. 7 (2023): 661–664. 37211511 10.1016/j.tim.2023.04.007

[99] J. A. Gilbert and E. M. Hartmann , “The Indoors Microbiome and Human Health,” Nature Reviews. Microbiology 22, no. 12 (2024): 742–755. 39030408 10.1038/s41579-024-01077-3

[100] G. A. W. Rook , V. Adams , J. Hunt , R. Palmer , R. Martinelli , and L. R. Brunet , “Mycobacteria and Other Environmental Organisms as Immunomodulators for Immunoregulatory Disorders,” Springer Seminars in Immunopathology 25, no. 3 (2004): 237–255. 15007629 10.1007/s00281-003-0148-9

[101] G. A. W. Rook , C. A. Lowry , and C. L. Raison , “Microbial ‘Old Friends’, Immunoregulation and Stress Resilience,” Evol Med Public Health 2013, no. 1 (2013): 46–64. 24481186 10.1093/emph/eot004 PMC3868387

[102] L. von Hertzen , I. Hanski , and T. Haahtela , “Natural Immunity,” EMBO Reports 12, no. 11 (2011): 1089–1093. 21979814 10.1038/embor.2011.195 PMC3207110

[103] M. Saarenpää , M. I. Roslund , N. Nurminen , et al., “Urban Indoor Gardening Enhances Immune Regulation and Diversifies Skin Microbiota – A Placebo‐Controlled Double‐Blinded Intervention Study,” Environment International 187 (2024): 108705. 38688234 10.1016/j.envint.2024.108705

[104] I. Hanski , L. von Hertzen , N. Fyhrquist , et al., “Environmental Biodiversity, Human Microbiota, and Allergy Are Interrelated,” National Academy of Sciences of the United States of America 109, no. 21 (2012): 8334–8339. 10.1073/pnas.1205624109 PMC3361383 22566627

[105] C. A. Akdis , “Does the Epithelial Barrier Hypothesis Explain the Increase in Allergy, Autoimmunity and Other Chronic Conditions?,” Nature Reviews. Immunology 21, no. 11 (2021): 739–751. 10.1038/s41577-021-00538-7 33846604

[106] S. Rakoff‐Nahoum , J. Paglino , F. Eslami‐Varzaneh , S. Edberg , and R. Medzhitov , “Recognition of Commensal Microflora by Toll‐Like Receptors Is Required for Intestinal Homeostasis,” Cell 118, no. 2 (2004): 229–241. 15260992 10.1016/j.cell.2004.07.002

[107] R. C. Taylor , P. Richmond , and J. W. Upham , “Toll‐Like Receptor 2 Ligands Inhibit TH2 Responses to Mite Allergen,” Journal of Allergy and Clinical Immunology 117, no. 5 (2006): 1148–1154. 16675345 10.1016/j.jaci.2006.02.014

[108] M. J. Ege , C. Bieli , R. Frei , et al., “Prenatal Farm Exposure Is Related to the Expression of Receptors of the Innate Immunity and to Atopic Sensitization in School‐Age Children,” Journal of Allergy and Clinical Immunology 117, no. 4 (2006): 817–823. 16630939 10.1016/j.jaci.2005.12.1307

[109] US EPA: Report on the Environment , “Indoor Air Quality; What Are the Trends in Indoor Air Quality and Their Effects on Human Health?” https://www.epa.gov/report‐environment/indoor‐air‐quality#note1.

[110] Y. Liang , D. Sengupta , M. J. Campmier , D. M. Lunderberg , J. S. Apte , and A. H. Goldstein , “Wildfire Smoke Impacts on Indoor Air Quality Assessed Using Crowdsourced Data in California,” National Academy of Sciences of the United States of America 118, no. 36 (2021): e2106478118. 10.1073/pnas.2106478118 PMC8433518 34465624

[111] P. M. Shrestha , J. L. Humphrey , E. J. Carlton , et al., “Impact of Outdoor Air Pollution on Indoor Air Quality in Low‐Income Homes During Wildfire Seasons,” International Journal of Environmental Research and Public Health 16, no. 19 (2019): 3535. 31546585 10.3390/ijerph16193535 PMC6801919

[112] M. T. Baeza‐Romero , M. R. Dudzinska , M. Amouei Torkmahalleh , et al., “A Review of Critical Residential Buildings Parameters and Activities When Investigating Indoor Air Quality and Pollutants,” Indoor Air 32, no. 11 (2022): e13144. 36437669 10.1111/ina.13144 PMC9828800

[113] S. Wang , C. X. Gao , J. Ye , X. Zheng , and H. Qian , “Indoor‐Outdoor Microbial Exchange: Quantifying the Contribution of Outdoor Sources to Indoor Microbiota Across Seasons,” Journal of Building Engineering 92 (2024): 109806.

[114] S. Wang , H. Qian , Z. Sun , G. Cao , P. Ding , and X. Zheng , “Comparison of Airborne Bacteria and Fungi in Different Built Environments in Selected Cities in Five Climate Zones of China,” Science of the Total Environment 860 (2023): 160445. 36436636 10.1016/j.scitotenv.2022.160445

[115] Y. Li , Y. Ge , C. Wu , D. Guan , J. Liu , and F. Wang , “Assessment of Culturable Airborne Bacteria of Indoor Environments in Classrooms, Dormitories and Dining Hall at University: A Case Study in China,” Aerobiologia (Bologna) 36, no. 3 (2020): 313–324. 32421086 10.1007/s10453-020-09633-z PMC7223800

[116] F. M. Chegini , A. N. Baghani , M. S. Hassanvand , et al., “Indoor and Outdoor Airborne Bacterial and Fungal Air Quality in Kindergartens: Seasonal Distribution, Genera, Levels, and Factors Influencing Their Concentration,” Building and Environment 175 (2020): 106690.

[117] L. Kazensky , K. Matković , M. Gerić , B. Žegura , G. Pehnec , and G. Gajski , “Impact of Indoor Air Pollution on DNA Damage and Chromosome Stability: A Systematic Review,” Archives of Toxicology 98, no. 9 (2024): 2817–2841. 38805047 10.1007/s00204-024-03785-4

[118] Y. Wāng , “Ambient Fine Particulate Matter Provokes Multiple Modalities of Cell Death via Perturbation of Subcellular Structures,” Environment International 195 (2025): 109193. 39721566 10.1016/j.envint.2024.109193

[119] G. Pizzino , N. Irrera , M. Cucinotta , et al., “Oxidative Stress: Harms and Benefits for Human Health,” Oxidative Medicine and Cellular Longevity 2017, no. 1 (2017): 8416763. 28819546 10.1155/2017/8416763 PMC5551541

[120] C. A. Juan , J. M. de la Lastra , F. J. Plou , and E. Pérez‐Lebeña , “The Chemistry of Reactive Oxygen Species (ROS) Revisited: Outlining Their Role in Biological Macromolecules (DNA, Lipids and Proteins) and Induced Pathologies,” International Journal of Molecular Sciences 22, no. 9 (2021): 4642. 33924958 10.3390/ijms22094642 PMC8125527

[121] K. Aramouni , R. Assaf , A. Shaito , et al., “Biochemical and Cellular Basis of Oxidative Stress: Implications for Disease Onset,” Journal of Cellular Physiology 238, no. 9 (2023): 1951–1963. 37436042 10.1002/jcp.31071

[122] P. Møller , P. H. Danielsen , D. G. Karottki , et al., “Oxidative Stress and Inflammation Generated DNA Damage by Exposure to Air Pollution Particles,” Mutation Research, Reviews in Mutation Research 762 (2014): 133–166. 25475422 10.1016/j.mrrev.2014.09.001

[123] E. Y. Lim and G. D. Kim , “Particulate Matter‐Induced Emerging Health Effects Associated With Oxidative Stress and Inflammation,” Antioxidants 13, no. 10 (2024): 1256. 39456509 10.3390/antiox13101256 PMC11505051

[124] Z. Leni , L. Künzi , and M. Geiser , “Air Pollution Causing Oxidative Stress,” Current Opinion in Toxicology 20‐21 (2020): 1–8.

[125] S. Viegas , C. Ladeira , A. Costa‐Veiga , J. Perelman , and G. Gajski , “Forgotten Public Health Impacts of Cancer – An Overview,” Archives of Industrial Hygiene and Toxicology 68, no. 4 (2017): 287–297. 29337686 10.1515/aiht-2017-68-3005

[126] Y. H. Lim , L. G. Hersoug , R. Lund , et al., “Inflammatory Markers and Lung Function in Relation to Indoor and Ambient Air Pollution,” International Journal of Hygiene and Environmental Health 241 (2022): 113944. 35176573 10.1016/j.ijheh.2022.113944

[127] M. Fandiño‐Del‐Rio , J. L. Kephart , K. N. Williams , et al., “Household Air Pollution and Blood Markers of Inflammation: A Cross‐Sectional Analysis,” Indoor Air 31, no. 5 (2021): 1509–1521. 33749948 10.1111/ina.12814 PMC8380676

[128] K. K. Lee , R. Bing , J. Kiang , et al., “Adverse Health Effects Associated With Household Air Pollution: A Systematic Review, Meta‐Analysis, and Burden Estimation Study,” Lancet Global Health 8, no. 11 (2020): e1427–e1434. 33069303 10.1016/S2214-109X(20)30343-0 PMC7564377

[129] M. Lovrić , G. Gajski , J. Fernández‐Agüera , et al., “Evidence Driven Indoor Air Quality Improvement: An Innovative and Interdisciplinary Approach to Improving Indoor Air Quality,” BioFactors 51, no. 1 (2025): e2126. 39350641 10.1002/biof.2126

[130] M. Fenech , “Cytokinesis‐Block Micronucleus Cytome Assay,” Nature Protocols 2, no. 5 (2007): 1084–1104. 17546000 10.1038/nprot.2007.77

[131] A. Collins , P. Møller , G. Gajski , et al., “Measuring DNA Modifications With the Comet Assay: A Compendium of Protocols,” Nature Protocols 18, no. 3 (2023): 929–989. 36707722 10.1038/s41596-022-00754-y PMC10281087

[132] G. Gajski , V. Kašuba , M. Milić , et al., “Exploring Cytokinesis Block Micronucleus Assay in Croatia: A Journey Through the Past, Present, and Future in Biomonitoring of the General Population,” Mutation Research, Genetic Toxicology and Environmental Mutagenesis 895 (2024): 503749. 38575251 10.1016/j.mrgentox.2024.503749

[133] C. Ladeira , P. Møller , L. Giovannelli , et al., “The Comet Assay as a Tool in Human Biomonitoring Studies of Environmental and Occupational Exposure to Chemicals – A Systematic Scoping Review,” Toxics 12, no. 4 (2024): 270. 38668493 10.3390/toxics12040270 PMC11054096

[134] S. Sommer , I. Buraczewska , and M. Kruszewski , “Micronucleus Assay: The State of Art, and Future Directions,” International Journal of Molecular Sciences 21, no. 4 (2020): 1534. 32102335 10.3390/ijms21041534 PMC7073234

[135] G. Gajski , S. Langie , and A. Zhanataev , “Recent Applications of the Comet Assay: A Report From the International Comet Assay Workshop 2019,” Toxicology Letters 333 (2020): 1–3. 32721575 10.1016/j.toxlet.2020.07.022

[136] A. Azqueta , C. Ladeira , L. Giovannelli , et al., “Application of the Comet Assay in Human Biomonitoring: An hCOMET Perspective,” Mutation Research, Reviews in Mutation Research 783 (2020): 108288. 32192646 10.1016/j.mrrev.2019.108288

[137] M. Milić , M. Ceppi , M. Bruzzone , et al., “The hCOMET Project: International Database Comparison of Results With the Comet Assay in Human Biomonitoring. Baseline Frequency of DNA Damage and Effect of Main Confounders,” Mutation Research, Reviews in Mutation Research 787 (2021): 108371. 34083035 10.1016/j.mrrev.2021.108371 PMC8525632

[138] A. Nersesyan , M. Mišík , A. Cherkas , et al., “Use of Micronucleus Experiments for the Detection of Human Cancer Risks: A Brief Overview,” Proceeding of the Shevchenko Scientific Society Medical Sciences 65, no. 2 (2021): 50–58.

[139] M. Gerić , G. Pehnec , K. Matković , et al., “Air Pollution and Primary DNA Damage Among Zagreb (Croatia) Residents: A Cross‐Sectional Study,” Journal of Xenobiotics 14, no. 1 (2024): 368–379. 38535498 10.3390/jox14010023 PMC10971122

[140] P. Møller , A. Azqueta , E. Boutet‐Robinet , et al., “Minimum Information for Reporting on the Comet Assay (MIRCA): Recommendations for Describing Comet Assay Procedures and Results,” Nature Protocols 15, no. 12 (2020): 3817–3826. 33106678 10.1038/s41596-020-0398-1 PMC7688437

[141] G. Gajski , M. Gerić , V. Oreščanin , and V. Garaj‐Vrhovac , “Cytokinesis‐Block Micronucleus Cytome Assay Parameters in Peripheral Blood Lymphocytes of the General Population: Contribution of Age, Sex, Seasonal Variations and Lifestyle Factors,” Ecotoxicology and Environmental Safety 148 (2018): 561–570. 29127818 10.1016/j.ecoenv.2017.11.003

[142] A. Nersesyan , M. Kundi , M. Fenech , et al., “Recommendations and Quality Criteria for Micronucleus Studies With Humans,” Mutation Research, Reviews in Mutation Research 789 (2022): 108410. 35690413 10.1016/j.mrrev.2021.108410

[143] S. Bonassi , A. Znaor , M. Ceppi , et al., “An Increased Micronucleus Frequency in Peripheral Blood Lymphocytes Predicts the Risk of Cancer in Humans,” Carcinogenesis 28, no. 3 (2007): 625–631. 16973674 10.1093/carcin/bgl177

[144] S. Bonassi , M. Ceppi , P. Møller , et al., “DNA Damage in Circulating Leukocytes Measured With the Comet Assay May Predict the Risk of Death,” Scientific Reports 11, no. 1 (2021): 16793. 34408182 10.1038/s41598-021-95976-7 PMC8373872

[145] M. R. Pandey , “Domestic Smoke Pollution and Chronic Bronchitis in a Rural Community of the Hill Region of Nepal,” Thorax 39, no. 5 (1984): 337. 6740536 10.1136/thx.39.5.337 PMC459798

[146] J. Groot , E. T. Nielsen , T. F. Nielsen , et al., “Exposure to Residential Mold and Dampness and the Associations With Respiratory Tract Infections and Symptoms Thereof in Children in High Income Countries: A Systematic Review and Meta‐Analyses of Epidemiological Studies,” Paediatric Respiratory Reviews 48 (2023): 47–64. 37482434 10.1016/j.prrv.2023.06.003

[147] R. Sauni , J. H. Verbeek , J. Uitti , M. Jauhiainen , K. Kreiss , and T. Sigsgaard , “Remediating Buildings Damaged by Dampness and Mould for Preventing or Reducing Respiratory Tract Symptoms, Infections and Asthma,” Cochrane Database of Systematic Reviews 2 (2015): CD007897. 10.1002/14651858.CD007897.pub3 PMC6769180 25715323

[148] I. A. Neamtiu , S. Lin , M. Chen , C. Roba , E. Csobod , and E. S. Gurzau , “Assessment of Formaldehyde Levels in Relation to Respiratory and Allergic Symptoms in Children From Alba County Schools, Romania,” Environmental Monitoring and Assessment 191, no. 9 (2019): 591. 31446497 10.1007/s10661-019-7768-6

[149] D. Norbäck , Z. Hashim , F. Ali , and J. H. Hashim , “Asthma Symptoms and Respiratory Infections in Malaysian Students‐Associations With Ethnicity and Chemical Exposure at Home and School,” Environmental Research 197 (2021): 111061. 33785322 10.1016/j.envres.2021.111061

[150] M. Takaoka , K. Suzuki , and D. Norbäck , “Current Asthma, Respiratory Symptoms and Airway Infections Among Students in Relation to the School and Home Environment in Japan,” Journal of Asthma 54, no. 6 (2017): 652–661. 10.1080/02770903.2016.1255957 28635545

[151] T. Taskinen , A. Hyvärinen , T. Meklin , T. Husman , A. Nevalainen , and M. Korppi , “Asthma and Respiratory Infections in School Children With Special Reference to Moisture and Mold Problems in the School,” Acta Paediatrica 88, no. 12 (1999): 1373–1379. 10626525 10.1080/080352599750030112

[152] T. Putus , A. Tuomainen , and S. Rautiala , “Chemical and Microbial Exposures in a School Building: Adverse Health Effects in Children,” Archives of Environmental Health: An International Journal 59, no. 4 (2004): 194–201. 10.3200/AEOH.59.4.194-201 16189992

[153] A. Borràs‐Santos , J. H. Jacobs , M. Täubel , et al., “Dampness and Mould in Schools and Respiratory Symptoms in Children: The HITEA Study,” Occupational and Environmental Medicine 70, no. 10 (2013): 681. 23775866 10.1136/oemed-2012-101286

[154] J. R. Palumbo , S. Lin , Z. Lin , et al., “Assessing Associations Between Indoor Environment and Health Symptoms in Romanian School Children: An Analysis of Data From the SINPHONIE Project,” Environmental Science and Pollution Research 25, no. 9 (2018): 9186–9193. 29473137 10.1007/s11356-018-1568-3

[155] Z. Lin , S. Lin , I. A. Neamtiu , et al., “Predicting Environmental Risk Factors in Relation to Health Outcomes Among School Children From Romania Using Random Forest Model – An Analysis of Data From the SINPHONIE Project,” Science of the Total Environment 784 (2021): 147145. 33901961 10.1016/j.scitotenv.2021.147145

[156] O. Toyinbo , M. Matilainen , M. Turunen , T. Putus , R. Shaughnessy , and U. Haverinen‐Shaughnessy , “Modeling Associations Between Principals' Reported Indoor Environmental Quality and Students' Self‐Reported Respiratory Health Outcomes Using GLMM and ZIP Models,” International Journal of Environmental Research and Public Health 13, no. 4 (2016): 385. 27043595 10.3390/ijerph13040385 PMC4847047

[157] R. T. Edwards , R. D. Neal , P. Linck , et al., “Enhancing Ventilation in Homes of Children With Asthma: Cost‐Effectiveness Study Alongside Randomised Controlled Trial,” British Journal of General Practice 61, no. 592 (2011): e733–e741. 10.3399/bjgp11X606645 PMC3207091 22054337

[158] M. Jutel , G. S. Mosnaim , J. A. Bernstein , et al., “The One Health Approach for Allergic Diseases and Asthma,” Allergy 78, no. 7 (2023): 1777–1793. 37119496 10.1111/all.15755

[159] L. Ruokolainen , L. von Hertzen , N. Fyhrquist , et al., “Green Areas Around Homes Reduce Atopic Sensitization in Children,” Allergy 70, no. 2 (2015): 195–202. 25388016 10.1111/all.12545 PMC4303942

[160] L. B. Murrison , E. B. Brandt , J. B. Myers , and G. K. K. Hershey , “Environmental Exposures and Mechanisms in Allergy and Asthma Development,” Journal of Clinical Investigation 129, no. 4 (2019): 1504–1515. 30741719 10.1172/JCI124612 PMC6436881

[161] M. S. Waring and J. R. Wells , “Volatile Organic Compound Conversion by Ozone, Hydroxyl Radicals, and Nitrate Radicals in Residential Indoor Air: Magnitudes and Impacts of Oxidant Sources,” Atmospheric Environment 106 (2015): 382–391. 26855604 10.1016/j.atmosenv.2014.06.062 PMC4741105

[162] C. J. Weschler , “Ozone's Impact on Public Health: Contributions From Indoor Exposures to Ozone and Products of Ozone‐Initiated Chemistry,” Environmental Health Perspectives 114, no. 10 (2006): 1489–1496. 17035131 10.1289/ehp.9256 PMC1626413

[163] K. Rumchev , J. Spickett , M. Bulsara , M. Phillips , and S. Stick , “Association of Domestic Exposure to Volatile Organic Compounds With Asthma in Young Children,” Thorax 59, no. 9 (2004): 746–751. 15333849 10.1136/thx.2003.013680 PMC1747137

[164] T. Jartti , K. Bønnelykke , V. Elenius , and W. Feleszko , “Role of Viruses in Asthma,” Seminars in Immunopathology 42, no. 1 (2020): 61–74. 31989228 10.1007/s00281-020-00781-5 PMC7066101

[165] I. Agache , C. Canelo‐Aybar , I. Annesi‐Maesano , et al., “The Impact of Indoor Pollution on Asthma‐Related Outcomes: A Systematic Review for the EAACI Guidelines on Environmental Science for Allergic Diseases and Asthma,” Allergy 79, no. 7 (2024): 1761–1788. 38366695 10.1111/all.16051

[166] J. V. Fahy , “Type 2 Inflammation in Asthma – Present in Most, Absent in Many,” Nature Reviews. Immunology 15, no. 1 (2015): 57–65. 10.1038/nri3786 PMC4390063 25534623

[167] C. J. Xu , C. Söderhäll , M. Bustamante , et al., “DNA Methylation in Childhood Asthma: An Epigenome‐Wide Meta‐Analysis,” Lancet Respiratory Medicine 6, no. 5 (2018): 379–388. 29496485 10.1016/S2213-2600(18)30052-3

[168] E. Forno , T. Wang , C. Qi , et al., “DNA Methylation in Nasal Epithelium, Atopy, and Atopic Asthma in Children: A Genome‐Wide Study,” Lancet Respiratory Medicine 7, no. 4 (2019): 336–346. 30584054 10.1016/S2213-2600(18)30466-1 PMC6441380

[169] E. Legaki , S. Taka , and N. G. Papadopoulos , “The Complexity in DNA Methylation Analysis of Allergic Diseases,” Current Opinion in Allergy and Clinical Immunology 23, no. 2 (2023): 172–178. 36752374 10.1097/ACI.0000000000000895

[170] T. Jartti and J. E. Gern , “Role of Viral Infections in the Development and Exacerbation of Asthma in Children,” Journal of Allergy and Clinical Immunology 140, no. 4 (2017): 895–906. 28987219 10.1016/j.jaci.2017.08.003 PMC7172811

[171] B. Jakiela , R. Brockman‐Schneider , S. Amineva , W. M. Lee , and J. E. Gern , “Basal Cells of Differentiated Bronchial Epithelium Are More Susceptible to Rhinovirus Infection,” American Journal of Respiratory Cell and Molecular Biology 38, no. 5 (2008): 517–523. 18063839 10.1165/rcmb.2007-0050OC PMC2358970

[172] K. Bønnelykke , A. T. Coleman , M. D. Evans , et al., “Cadherin‐Related Family Member 3 Genetics and Rhinovirus C Respiratory Illnesses,” American Journal of Respiratory and Critical Care Medicine 197, no. 5 (2017): 589–594. 10.1164/rccm.201705-1021OC PMC6005238 29121479

[173] F. J. Rubner , D. J. Jackson , M. D. Evans , et al., “Early Life Rhinovirus Wheezing, Allergic Sensitization, and Asthma Risk at Adolescence,” Journal of Allergy and Clinical Immunology 139, no. 2 (2017): 501–507. 27312820 10.1016/j.jaci.2016.03.049 PMC5104680

[174] M. Calışkan , Y. A. Bochkov , E. Kreiner‐Møller , et al., “Rhinovirus Wheezing Illness and Genetic Risk of Childhood‐Onset Asthma,” New England Journal of Medicine 368, no. 15 (2024): 1398–1407. 10.1056/NEJMoa1211592 PMC3755952 23534543

[175] M. Lukkarinen , A. Koistinen , R. Turunen , P. Lehtinen , T. Vuorinen , and T. Jartti , “Rhinovirus‐Induced First Wheezing Episode Predicts Atopic but Not Nonatopic Asthma at School Age,” Journal of Allergy and Clinical Immunology 140, no. 4 (2017): 988–995. 28347734 10.1016/j.jaci.2016.12.991 PMC7172285

[176] A. Wrotek , A. Badyda , P. O. Czechowski , T. Owczarek , P. Dąbrowiecki , and T. Jackowska , “Air Pollutants' Concentrations Are Associated With Increased Number of RSV Hospitalizations in Polish Children,” Journal of Clinical Medicine 10, no. 15 (2021): 3224. 34362009 10.3390/jcm10153224 PMC8348891

[177] V. Peltola , M. Waris , R. Österback , P. Susi , O. Ruuskanen , and T. Hyypiä , “Rhinovirus Transmission Within Families With Children: Incidence of Symptomatic and Asymptomatic Infections,” Journal of Infectious Diseases 197, no. 3 (2008): 382–389. 18248302 10.1086/525542

[178] S. Gilles , C. Blume , M. Wimmer , et al., “Pollen Exposure Weakens Innate Defense Against Respiratory Viruses,” Allergy 75, no. 3 (2020): 576–587. 31512243 10.1111/all.14047

[179] I. Beck , S. Jochner , S. Gilles , et al., “High Environmental Ozone Levels Lead to Enhanced Allergenicity of Birch Pollen,” PLoS One 8, no. 11 (2013): e80147. 24278250 10.1371/journal.pone.0080147 PMC3835901

[180] Z. Celebi Sozener , B. Ozdel Ozturk , P. Cerci , et al., “Epithelial Barrier Hypothesis: Effect of the External Exposome on the Microbiome and Epithelial Barriers in Allergic Disease,” Allergy 77, no. 5 (2022): 1418–1449. 35108405 10.1111/all.15240 PMC9306534

[181] N. Sun , I. Ogulur , Y. Mitamura , et al., “The Epithelial Barrier Theory and Its Associated Diseases,” Allergy 79, no. 12 (2024): 3192–3237. 39370939 10.1111/all.16318 PMC11657050

[182] I. Agache , C. Akdis , M. Akdis , et al., “Immune‐Mediated Disease Caused by Climate Change‐Associated Environmental Hazards: Mitigation and Adaptation,” Frontiers in Science 2 (2024): 1279192. 40444110 10.3389/fsci.2024.1279192 PMC12121949

[183] T. Chen , S. Shi , X. Li , et al., “Improved Ambient Air Quality Is Associated With Decreased Prevalence of Childhood Asthma and Infancy Shortly After Weaning Is a Sensitive Exposure Window,” Allergy 79, no. 5 (2024): 1166–1179. 37458141 10.1111/all.15815

[184] B. Moorthy , C. Chu , and D. J. Carlin , “Polycyclic Aromatic Hydrocarbons: From Metabolism to Lung Cancer,” Toxicological Sciences 145, no. 1 (2015): 5–15. 25911656 10.1093/toxsci/kfv040 PMC4408964

[185] A. J. Badyda , W. Rogula‐Kozłowska , G. Majewski , et al., “Inhalation Risk to PAHs and BTEX During Barbecuing: The Role of Fuel/Food Type and Route of Exposure,” Journal of Hazardous Materials 440 (2022): 129635. 36027742 10.1016/j.jhazmat.2022.129635

[186] L. Wu , X. Lu , S. Zhang , et al., “Co‐Exposure Effects of Urinary Polycyclic Aromatic Hydrocarbons and Metals on Lung Function: Mediating Role of Systematic Inflammation,” BMC Pulmonary Medicine 24, no. 1 (2024): 386. 39128985 10.1186/s12890-024-03173-9 PMC11316979

[187] M. Låg , J. Øvrevik , M. Refsnes , and J. A. Holme , “Potential Role of Polycyclic Aromatic Hydrocarbons in Air Pollution‐Induced Non‐Malignant Respiratory Diseases,” Respiratory Research 21, no. 1 (2020): 299. 33187512 10.1186/s12931-020-01563-1 PMC7666487

[188] A. Chciałowski , P. Dąbrowiecki , D. Jaskóła‐Polkowska , A. Rzeszotarska , and J. Korsak , “Particulate Matter and Polycyclic Aromatic Hydrocarbons Influence on Respiratory Function and the Possibility of Allergy in Healthy Adults,” Austin Environmental Sciences 9, no. 1 (2024): id1103.

[189] P. Dąbrowiecki , A. Chciałowski , A. Dąbrowiecka , and A. Badyda , “Ambient Air Pollution and Risk of Admission due to Asthma in the Three Largest Urban Agglomerations in Poland: A Time‐Stratified, Case‐Crossover Study,” International Journal of Environmental Research and Public Health 19, no. 10 (2022): 5988. 35627528 10.3390/ijerph19105988 PMC9140383

[190] P. Dąbrowiecki , A. Chciałowski , A. Dąbrowiecka , A. Piórkowska , and A. Badyda , “Air Pollution and Long‐Term Risk of Hospital Admission due to Chronic Obstructive Pulmonary Disease Exacerbations in Poland: A Time‐Stratified, Case‐Crossover Study,” Polish Archives of Internal Medicine 133, no. 7‐8 (2023): 16444. 36856604 10.20452/pamw.16444

[191] A. R. Ferro , R. J. Kopperud , and L. M. Hildemann , “Elevated Personal Exposure to Particulate Matter From Human Activities in a Residence,” Journal of Exposure Science & Environmental Epidemiology 14, no. 1 (2004): S34–S40. 10.1038/sj.jea.7500356 15118743

[192] J. C. Rufo , I. Annesi‐Maesano , P. Carreiro‐Martins , et al., “Issue 2 – “Update on Adverse Respiratory Effects of Indoor Air Pollution” Part 1: Indoor Air Pollution and Respiratory Diseases: A General Update and a Portuguese Perspective,” Pulmonology 30, no. 4 (2024): 378–389. 37230882 10.1016/j.pulmoe.2023.03.006

[193] M. Bentayeb , C. Billionnet , N. Baiz , M. Derbez , S. Kirchner , and I. Annesi‐Maesano , “Higher Prevalence of Breathlessness in Elderly Exposed to Indoor Aldehydes and VOCs in a Representative Sample of French Dwellings,” Respiratory Medicine 107, no. 10 (2013): 1598–1607. 23920330 10.1016/j.rmed.2013.07.015

[194] M. U. Ali , Y. Yu , B. Yousaf , et al., “Health Impacts of Indoor Air Pollution From Household Solid Fuel on Children and Women,” Journal of Hazardous Materials 416 (2021): 126127. 34492921 10.1016/j.jhazmat.2021.126127

[195] J. Sunyer , M. Esnaola , M. Alvarez‐Pedrerol , et al., “Association Between Traffic‐Related Air Pollution in Schools and Cognitive Development in Primary School Children: A Prospective Cohort Study,” PLoS Medicine 12, no. 3 (2015): e1001792. 25734425 10.1371/journal.pmed.1001792 PMC4348510

[196] J. Forns , P. Dadvand , M. Esnaola , et al., “Longitudinal Association Between Air Pollution Exposure at School and Cognitive Development in School Children Over a Period of 3.5 Years,” Environmental Research 159 (2017): 416–421. 28858754 10.1016/j.envres.2017.08.031

[197] E. Midouhas , T. Kokosi , and E. Flouri , “Outdoor and Indoor Air Quality and Cognitive Ability in Young Children,” Environmental Research 161 (2018): 321–328. 29182908 10.1016/j.envres.2017.11.026

[198] M. Kuntić , O. Hahad , T. Münzel , and A. Daiber , “Crosstalk Between Oxidative Stress and Inflammation Caused by Noise and Air Pollution – Implications for Neurodegenerative Diseases,” Antioxidants 13, no. 3 (2024): 266. 38539800 10.3390/antiox13030266 PMC10967531

[199] M. Thygesen , G. J. Holst , B. Hansen , et al., “Exposure to Air Pollution in Early Childhood and the Association With Attention‐Deficit Hyperactivity Disorder,” Environmental Research 183 (2020): 108930. 31810593 10.1016/j.envres.2019.108930 PMC7167333

[200] A. E. Margolis , J. B. Herbstman , K. S. Davis , et al., “Longitudinal Effects of Prenatal Exposure to Air Pollutants on Self‐Regulatory Capacities and Social Competence,” Journal of Child Psychology and Psychiatry 57, no. 7 (2016): 851–860. 26989990 10.1111/jcpp.12548 PMC5333974

